# Prevalence and prognostic value of neurological affections in hospitalized patients with moderate to severe COVID-19 based on objective assessments

**DOI:** 10.1038/s41598-023-46124-w

**Published:** 2023-11-10

**Authors:** Carolin Balloff, Carolina Bandlow, Michael Bernhard, Timo Brandenburger, Patricia Bludau, Saskia Elben, Torsten Feldt, Christian J. Hartmann, Elisa Heinen, Jens Ingwersen, Corinna Jansen, Björn-Erik O. Jensen, Detlef Kindgen-Milles, Tom Luedde, Iris-Katharina Penner, Isabel Slink, Kim Stramm, Ann-Kathrin Telke, Jörg Timm, Lana Vetterkind, Christian Vollmer, Georg Wolff, Alfons Schnitzler, Sven G. Meuth, Stefan J. Groiss, Philipp Albrecht

**Affiliations:** 1 Department of Neurology, Medical Faculty, Heinrich-Heine-University, 40225 Duesseldorf, Germany; 2 Department of Neurology, Kliniken Maria Hilf GmbH, 41063 Moenchengladbach, Germany; 3 Emergency Department, Medical Faculty, Heinrich-Heine-University, 40225 Duesseldorf, Germany; 4 Department of Anesthesiology, Medical Faculty, Heinrich-Heine-University, 40225 Duesseldorf, Germany; 5 Department of Gastroenterology, Hepatology and Infectious Diseases, Medical Faculty, Heinrich-Heine-University, 40225 Duesseldorf, Germany; 6 Institute of Clinical Neuroscience and Medical Psychology, Medical Faculty, Heinrich-Heine-University, 40225 Duesseldorf, Germany; 7 Department of Neurology, Inselspital, Bern University Hospital, University of Bern, 3010 Bern, Switzerland; 8 Department of Virology, Medical Faculty, Heinrich-Heine-University, 40225 Duesseldorf, Germany; 9 Department of Cardiology, Pulmonology, and Vascular Medicine, Medical Faculty, Heinrich-Heine-University, 40225 Duesseldorf, Germany; 10 Neurocenter Duesseldorf, 40211 Duesseldorf, Germany

**Keywords:** Medical research, Neurology, Risk factors, Signs and symptoms

## Abstract

Neurological manifestations of coronavirus disease 2019 (COVID-19) have been frequently described. In this prospective study of hospitalized COVID-19 patients without a history of neurological conditions, we aimed to analyze their prevalence and prognostic value based on established, standardized and objective methods. Patients were investigated using a multimodal electrophysiological approach, accompanied by neuropsychological and neurological examinations. Prevalence rates of central (CNS) and peripheral (PNS) nervous system affections were calculated and the relationship between neurological affections and mortality was analyzed using Firth logistic regression models. 184 patients without a history of neurological diseases could be enrolled. High rates of PNS affections were observed (66% of 138 patients receiving electrophysiological PNS examination). CNS affections were less common but still highly prevalent (33% of 139 examined patients). 63% of patients who underwent neuropsychological testing (*n* = 155) presented cognitive impairment. Logistic regression models revealed pathology in somatosensory evoked potentials as an independent risk factor of mortality (Odds Ratio: 6.10 [1.01–65.13], *p* = 0.049). We conclude that hospitalized patients with moderate to severe COVID-19 display high rates of PNS and CNS affection, which can be objectively assessed by electrophysiological examination. Electrophysiological assessment may have a prognostic value and could thus be helpful to identify patients at risk for deterioration.

## Introduction

Since the first outbreak of coronavirus disease 2019 (COVID-19) caused by the severe acute respiratory syndrome coronavirus-2 (SARS-CoV-2), affections of the human central (CNS) and peripheral nervous system (PNS) have been repeatedly described. The most frequently encountered neurological manifestations are fatigue, myalgia, taste and/or smell impairment and headache^1^.

A recent meta-analysis reported at least one neurological manifestation in one-third of patients with COVID-19 and an association with mortality in patients ≥ 60 years^1^. In line with this, an individual patient data (IPD) meta-analysis on hospitalized COVID-19 patients with neurological complications revealed that these patients were more likely to die within 30 days than hospitalized COVID-19 patients in general^2^. Importantly, neurological complications may not only manifest as overt symptoms, but also as subtle presentations^3^.

However, some studies included patients with preexisting neurological conditions^4–8^, did not report how previous neurological diseases were handled^9^,⁠ or only included patients with neurological affections^2^. Therefore, conclusions regarding SARS-CoV-2 specific effects are limited. Further, definition of and diagnostic criteria for neurological manifestations in COVID-19 oftentimes remain unreported and are not standardized^1^.⁠ Lastly, some studies rely on patient-reported outcomes only^10,11^. Electrophysiological characterization of both PNS and CNS function to objectify the reported symptoms and to detect subclinical symptoms in larger cohorts is still missing.

Evoked potentials and nerve conduction studies (NCS) including the blink reflex (BR) as a marker of brainstem function and the sympathetic skin response (SSR) as a marker of autonomous nervous system (ANS) function represent well-established, non-invasive methods to objectively investigate neuronal function. In a case series we found that these measures, as well as neuropsychological outcomes, can be markedly impaired during and directly after COVID-19^12^. ⁠In line with this, a recent study found subclinical abnormalities in NCS two to six months after pneumonia due to SARS-CoV-2^13^.

In this study, we extended our multimodal approach to a large cohort of previously neurologically healthy hospitalized patients with COVID-19, which we investigated during the acute infection. Early identification of critical patients is crucial to guide clinical decision making and allocation of limited resources. We therefore aimed to analyze the extent and prevalence of neurological deficits using established, standardized and objective methods and to identify potential biomarkers of prognostic value. Since there is no research on the prevalence rates of CNS and PNS affections based on standard electrophysiological assessments (EA) yet, analyses were of exploratory nature.

## Methods

### Participants

Hospitalized patients with COVID-19 were recruited between May 2020 and March 2022 at the University Hospital Duesseldorf, Germany, to participate in the PROGNOSE study. Inclusion criterion was an ongoing infection with SARS-CoV-2, confirmed by real-time reverse-transcription polymerase chain reaction. Here, we only focus on patients with symptoms of COVID-19. Exclusion criteria were: (1) pregnancy, (2) previous or ongoing neurological conditions with possible influence on the study readouts, and (3) age < 18 years.

Patients with neurological preconditions that only affected some assessments (e.g., dementia) were excluded only for confounded investigations (e.g., neuropsychology). Study participation did not influence the clinical treatment, which was performed according to the best medical care available at the time of examination.

### Ethics approval and consent to participate

The study was approved by the ethical committee of the medical faculty of the Heinrich-Heine-University Düsseldorf (Study-Number 2020-979) and carried out in accordance with the declaration of Helsinki. Informed written consent was provided prior to participation by the patient or, in case of inability to consent, by relatives and post-hoc by the patient.

### Neurological and clinical assessment and laboratory markers

Since there is no COVID-19 specific score for classification of neurological symptoms and disability yet, the following established disability scores were adjusted to the COVID-19 pathology and determined by neurological examination: (1) Expanded Disability Status Scale (EDSS) based on the following Functional Systems (FS): brainstem, pyramidal, cerebral, cerebellar, sensory^14^, (2) Modified Rankin Scale^15^, (3) INCAT disability score^16^, (4) Barthel Index^17^. All scores are described in detail in the Supplementary Methods. The clinical status of the patient at the time of examination was assessed by the WHO clinical progression scale (WHO score), documenting disease severity from 0 (uninfected) to 10 (dead)^18^.

Blood samples were collected as part of the clinical routine during or shortly after admission, and the following laboratory markers were analyzed: C-reactive protein, urea, lymphocytes, procalcitonin, troponin, ferritin, lactate dehydrogenase, and D-dimers. At admission, the level of consciousness was assessed by the Glasgow Coma Scale^19^.

### Neuropsychological assessment

The neuropsychological assessment consisted of the Montreal Cognitive Assessment (MoCA, version 7)^20^ as a screening battery for mild cognitive impairment and the Symbol Digit Modalities Test (SDMT)^21^ as a measure of information processing speed (IPS). Delirium was assessed using the 4 ‘A’s Test^22^ and Confusion Assessment Method for use in intensive care unit (ICU) patients^23^. MoCA and SDMT scores were transformed into demographically adjusted z-scores (see Supplementary Methods)^24,25^. In case of language barriers, neuropsychological assessment was limited to the SDMT or cancelled.

### Electrophysiological assessment

The EA included NCS of the right tibial, sural and ulnar nerves, BR of the bilateral ocular orbicular muscle, SSR, and motor and somatosensory evoked potentials (MEP/SSEP) to/from all extremities. If the right side could not be assessed in the NCS (e.g., due to an intravenous line) or patients specifically reported symptoms on the left side requiring clinical examination, the left side was measured instead. MEP were recorded from bilateral tibialis anterior and 1st dorsal interosseus muscles. Supramaximal stimuli of bilateral medial and tibial nerves with at least 200 averages were used for SSEP, recording responses at the poplitea/Erb’s point, C5/T12 and Cp/Cz, respectively.

All measurements were carried out with a Nihon Kohden Neuropack X1 (Nihon Kohden Corporation, Tokyo, Japan) and Ag–AgCl surface electrodes (28 × 20 mm [MEP, SSR, and NCS]/ 20 × 15 mm [BR], Ambu, Ballerup, Denmark) and subdermal needle electrodes (SSEP/12 × 0.4 mm, Ambu, Ballerup, Denmark) were used for recordings. MEP were evoked by single pulse transcranial magnetic stimulation via a standard circular coil (90 mm outer diameter, The Magstim Company Ltd., Whitland, UK) connected to a Magstim 200 (The Magstim Company Ltd., Whitland, UK).

All EA were evaluated based on the clinical norms of the University Hospital Düsseldorf (Supplementary Tables S1–S5) and affections were classified into PNS, CNS, and ANS (multiple selection possible).

PNS affection was defined as any abnormality in the NCS (distal motor latency [DML], F-wave latency, compound muscle action potential [CMAP], sensory nerve action potential [SNAP], motor/sensory conduction velocity [mCV/sCV]) or the following abnormalities in the BR: (1) R1, iR2 and cR2 exclusively delayed on one side, or (2) R1 and iR2 delayed on one side and cR2 delayed on the other side. Axonal pathology was defined as a reduction in CMAP/SNAP amplitude, whereas demyelinating pathology was defined as a reduction in mCV/sCV or prolongation of DML or F-wave latency.

CNS affection was defined as (1) reduced N20 and/or P40 in the SSEP and normal peripheral response (defined as normal N10 in the SSEP, if available, or as normal latency measured in the NCS), (2) increased central motor conduction time (CMCT) in the MEP, (3) increased cortical latency in the MEP and normal peripheral response (measured in the NCS), (4) delayed R1 exclusively on one side in the BR, (5) delayed R2 exclusively on one side in the BR, 6) delayed R2 on both sides in the BR.

ANS affection was defined as pathological latencies in the SSR. Please refer to Supplementary Tables S1–S5 for applied cut-offs for each assessment.

### Statistical analyses

Since the primary goal of the study was to investigate the unknown prevalence of (sub)clinical neurological affections, sample size was based on the number of patients willing to participate rather than statistical power calculation.

Clinical and demographic differences between surviving and deceased patients were assessed by Fisher’s exact test for categorical data and Mann–Whitney-U-test for continuous variables with non-normal distribution. *P*-values < 0.05 were considered significant. Prevalence rates were calculated using crosstabulations (1) considering all patients, including those with missing data, and (2) including only patients who underwent the respective assessments. Exploratively, statistical analyses were repeated excluding patients with diabetes mellitus (DM) as the most prevalent potential confounding factor.

The relationship between abnormalities in the EA and death was assessed by Firth logistic regression models. Firth logistic regression models were also calculated for all predictive parameters for the patient’s outcome of the 4C Deterioration Model and 4C Mortality Score^26,27^, as well as for the WHO score^18^, sedation, and the Modified Rankin Scale^15^. Due to the exploratory nature of the study, we did not correct for multiple testing.

To avoid confounding influences of sedation, regression analyses were repeated excluding sedated patients. Further, age and sex were included as potentially confounding factors. All analyses were repeated using the raw data of the EA as independent factor.

Probabilities of mortality were estimated using Kaplan–Meier analysis and Cox proportional hazard models were used to compare the probability of death between patients with and without pathological findings in the EA. Again, analyses were conducted separately for the whole sample and, subsequently, only for non-sedated patients.

All analyses were conducted using R Studio (version 2021.09.1 + 372), except for Kaplan–Meier analysis and Cox proportional hazard models which were conducted using IBM SPSS Statistics (version 26).

## Results

### Sample characteristics

**Figure 1:**
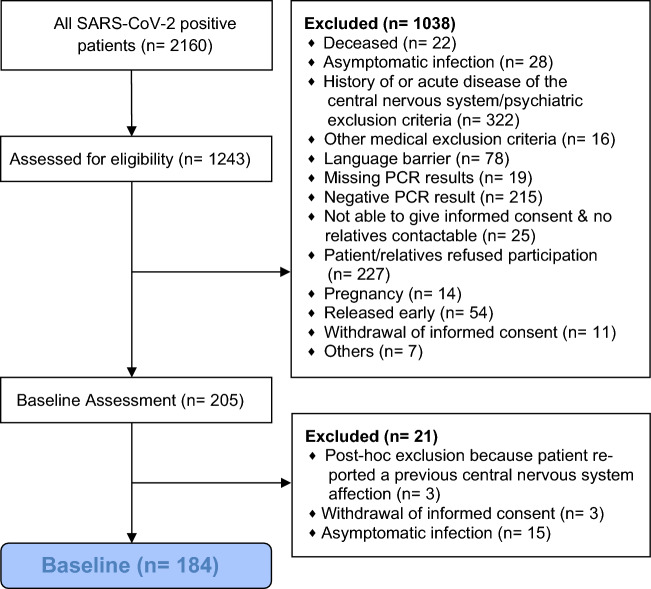
Flowchart of the enrollment of participants. The flowchart presents the number of participants at each step of the study. SARS-CoV-2, severe acute respiratory syndrome coronavirus-2; PCR, real-time reverse-transcription polymerase chain reaction.

Out of 1243 SARS-CoV-2 positive patients who were assessed for eligibility at the University Hospital Duesseldorf, 184 with definite COVID-19 and without a history of neurological diseases could be enrolled in the study (Fig. 1). Descriptive statistics are presented in Table 1 and individual data for each assessment are provided in Supplementary Figure S1. Compared to surviving patients, patients who died were significantly older, treated longer in the hospital, required ICU treatment and oxygen therapy more frequently, were more often comatose and more severely ill at the time of examination, as indicated by significantly higher WHO scores^18^. On average, they were also examined two days later after admission. Oxygen saturation and lymphocyte counts at admission were higher in survivors, while urea, C-reactive protein, procalcitonin, lactate dehydrogenase, and D-dimer concentrations were higher in the deceased group. More deceased than surviving patients suffered from the original virus variant.

**Table 1:** Clinical and demographic characteristics of the sample.

Characteristic	Survival (*N* = 170)	Death (*N* = 14)	*p *value
Age (years, median [IQR])	54.5 [24.6]	65.6 [15.3]	**0**.**0076**
Sex at birth (male, *N* [%])	112 [65.9]	12 [85*.*7]	0.15
ICU treatment required (yes, *N* [%])^a^	13 [7.6]	12 [85*.*7]	** < 0**.**0001**
Virus variant			**0**.**00021**
Original (*N* [%])	46 [27.1]	11 [78*.*6]	
B1.1.7 (alpha, *N* [%])	31 [18.2]	0 [0]	
B1.351 (beta, *N* [%])	0 [0]	1 [7*.*1]	
B.1.617 (delta, *N* [%])^b^	29 [17.1]	0 [0]	
B.1.1.529 (omikron, *N* [%])	28 [16.5]	1 [7*.*1]	
Unknown (*N* [%])	36 [21.2]	1 [7*.*1]	
Vaccination status (*N* [%])^c^			0.19
No vaccination	109 [64.1]	13 [92.9]	
Incomplete vaccination	5 [2.9]	0 [0]	
Complete vaccination	54 [31.8]	1 [7.1]	
Complete vaccination and booster	1 [0.6]	0 [0]	
Total days in hospital (days, median [IQR])	7 [7]	22.5 [19.6]	** < 0**.**0001**
Delay between hospitalization and examination (days, median [IQR])	3 [3]	5 [3]	**0**.**0018**
Delay between first positive PCR test and examination (days, median [IQR])^d^	4 [5]	5 [3]	0.43
Delay between symptom onset and examination (days, median [IQR])^e^	8 [6]	9 [4]	0.46
Coma (yes, *N* [%])	1 [5.9]	6 [42.9]	** < 0**.**0001**
Nosocomial infection (yes, *N* [%])^f^	3 [1.8]	0 [0]	> 0.99
Number of comorbidities (median [IQR])^g^	1 [2]	2 [1.8]	0.20
Radiographic infiltrates at admission (yes, *N* [%])^h^	97 [57.1]	10 [71.4]	0.25
Clinical scores at examination (median [IQR])			
EDSS^i^	1 [1.5]	1 [1.3]	0.39
MRS^j^	1 [0]	NA	NA
Barthel scale^k^	100 [5]	NA	NA
WHO clinical progression scale^l^	4 [1]	6 [3]	** < 0**.**0001**
INCAT disability score (overall)^m^	0 [0]	NA	NA
MRS at discharge (median [IQR])^n^	1 [1]	NA	NA
Education (years, median [IQR])^o^	13.8 [6.0]	NA	NA
MoCA total score^p^ (median [IQR])	− 1.78 [0.81]	NA	NA
SDMT z-score^q^ (median [IQR])	− 1.47 [− 1.67]	NA	NA
Vital parameters and blood results at admission			
Glasgow Coma Scale (median [IQR])^r^	15 [0]	15 [0]	> 0.99
Oxygen saturation (%, median [IQR])^s^	96 [3]	94 [15.5]	**0**.**0071**
Oxygen therapy (yes, *N* [%])^t^	48 [28.2]	8 [57.1]	**0**.**037**
Respiratory rate (breaths/min, median [IQR])^u^	18 [7]	17 [12]	0.89
Urea (md/dl, (median [IQR])	30 [21]	54 [45.8]	**0**.**0044**
C-reactive protein (mg/dl, median [IQR])	4.3 [7.6]	10.1 [13.9]	**0**.**0094**
Lymphocytes (× 1000/l, median [IQR])^v^	1.06 [0.92]	0.62 [0.25]	**0**.**0071**
Procalcitonin (ng/ml, median [IQR])^w^	0.10 [0.12]	0.85 [1.64]	**0**.**00044**
Troponin (ng/l, median [IQR])^x^	NA	19 [58]	NA
Ferritin (μg/l, median [IQR])^y^	631.5 [694.5]	724 [998.5]	0.73
Lactate Dehydrogenase (U/l, median [IQR])	307 [155.8]	466.5 [333.3]	**0**.**017**
D-Dimers (mg/l_FEU, median [IQR])^z^	0.73 [0.76]	2.04 [1.78]	**0**.**0010**

If more than half of the data are missing for one group, data are not displayed (not applicable). ICU, Intensive care unit; EDSS, Expanded Disability Status Scale; MRS, Modified Rankin Scale; MoCA, Montreal Cognitive Assessment; SDMT, Symbol Digit Modalities Test; NA = not applicable.

^a^*N*_Death_ = 11 & *N*_survival_ = 10 were treated at the ICU at the time of examination; *N*_Death_ = 1 & *N*_survival_ = 3 required ICU treatment after the examination.

^b^Including the following subspecies: AY.33 (*N* = 1), AY46.6 (*N* = 2), AY.44 (*N* = 1), AY.122 (*N* = 2), AY.43 (*N* = 2), AY.9 (*N* = 1), AY.126 (*N* = 1).

^c^Missing as follows: *N*_survival_ = 1.

^d^Missing as follows: *N*_Death_ = 2, *N*_survival_ = 8.

^e^Missing as follows: *N*_Death_ = 4, *N*_survival_ = 18.

^f^Missing as follows: *N*_Death_ = 2, *N*_survival_ = 50.

^g^Defined according to the definition implemented in the 4C Deterioration model^26^.

^h^Missing as follows: *N*_Death_ = 1, *N*_survival_ = 3.

^i^Missing as follows: *N*_Death_ = 7, *N*_survival_ = 15.

^j^Missing as follows: *N*_Death_ = 10, *N*_survival_ = 48.

^k^Missing as follows: *N*_Death_ = 10, *N*_survival_ = 49.

^l^Missing as follows: *N*_Death_ = 1.

^m^Missing as follows: *N*_Death_ = 8, *N*_survival_ = 47.

^n^Missing as follows: *N*_survival_ = 56.

^o^Defined as the total years in school and years of professional education (rounded to full years and not counting repeated years). The maximum was set at 20 years. Missing as follows: *N*_Death_ = 8, *N*_survival_ = 11.

^p^Missing as follows: *N*_Death_ = 8, *N*_survival_ = 41.

^q^Missing as follows: *N*_Death_ = 9, *N*_survival_ = 18.

^r^Missing as follows: *N*_survival_ = 1.

^s^Missing as follows: *N*_Death_ = 3, *N*_survival_ = 20.

^t^Missing as follows: *N*_survival_ = 4.

^u^Missing as follows: *N*_Death_ = 5, *N*_survival_ = 31.

^v^Missing as follows: *N*_Death_ = 3, *N*_survival_ = 48.

^w^Missing as follows: *N*_Death_ = 2, *N*_survival_ = 38.

^x^Missing as follows: *N*_Death_ = 1, *N*_survival_ = 101.

^y^Missing as follows: *N*_Death_ = 2, *N*_survival_ = 76.

^z^Missing as follows: *N*_Death_ = 1, *N*_survival_ = 66.

### Prevalence rates of abnormalities

Table 2 displays the neurological characteristics of all patients. PNS pathology occurred in a majority (66%) of all patients that underwent EA with slightly more sensible than motor affections (sensible: 59%, motor: 54%) of both axonal and demyelinating nature (axonal: 58%, demyelinating; 58%). Excluding patients with DM, prevalence rates of PNS affection remained equally high (64%, Supplementary Figure S2). Also taking into account patients who did not receive EA, the prevalence rate remained as high as 50%.

**Table 2:** Neurological characteristics.

Clinical findings (N, %)^a^	Electrophysiological and neuropsychological assessment							
	Central affection		Peripheral affection		Autonomous affection		Cognitive affection	
	Pathology	No Pathology	Pathology	No Pathology	Pathology	No Pathology	Pathology	No Pathology
All patients (N = 184, 100%)^b^	46 (25.0%_Total,_ 33.6%_CNS_)	91 (49.5%_Total,_ 66.4%_CNS_)	91 (49.5%_Total,_ 66.9%_PNS_)	45 (24.5%_Total,_ 33.1%_PNS_)	29 (15.8%_Total,_ 25.0%_ANS_)	87 (47.3%_Total,_ 75.0%_ANS_)	99 (53.8%_Total,_ 63.1%_Cog_)	58(31.5%_Total,_ 36.9%_Cog_)
Patients treated in ICU (N = 21, 11.4%)^c^	12 (57.1%_ICU,_ 60%_CNS_)	8 (38.1%_ICU,_ 40%_CNS_)	18 (85.7%_ICU,_ 90%_PNS_)	2 (9.5%_ICU,_ 10%_PNS_)	10 (47.6%_ICU,_ 55.6%_ANS_)	8(38.1%_ICU,_ 44.4%_ANS_)	9 (42.9%_ICU,_ 75.0%_Cog_)	3 (14.3%_ICU,_ 25.0%_Cog_)
Patients not treated in ICU (N = 163, 88.6%)^c^	34 (20.9%_NoICU,_ 29.1%_CNS_)	83 (50.9%_NoICU,_ 70.9%_CNS_)	73 (44.8%_NoICU,_ 62.9%_PNS_)	43 (26.4%_NoICU,_ 37.1%_PNS_)	19 (11.7%_NoICU,_ 19.4%_ANS_)	79 (48.5%_NoICU,_ 80.6%_ANS_)	90 (55.2%_NoICU,_ 62.1%_Cog_)	55 (33.7%_NoICU,_ 37.9%_Cog_)
Comatose/sedated patients (N = 8, 4.3%)	5 (62.5%_Total_, 62.5%_CNS_)	3 (37.5%_Total_, 37.5%_CNS_)	7 (87.5%_Total_, 87.5%_PNS_)	1 (12.5%_Total_, 12.5%_PNS_)	7 (87.5%_Total_, 100%_ANS_)	NA	NA	NA
Patients with affections in the functional systems^d^								
Any (N = 162)^e^								
Yes (N = 76, 41.3%_ Total,_ 46.9%_EDSS_)	N = 16	N = 42	N = 38	N = 20	N = 9	N = 40	N = 48	N = 25
No (N = 86, 46.7%_Total,_ 53.1%_EDSS_)	N = 20	N = 47	N = 40	N = 26	N = 10	N = 44	N = 39	N = 32
Brainstem (N = 164)^f^	Measured by BR							
Yes (N = 32, 17.4%_Total,_ 19.5%_FSS_)	N = 0	N = 24	N = 1	N = 23	NA	NA	NA	NA
No (N = 132, 71.7%_Total,_ 80.5%_FSS_)	N = 10	N = 85	N = 2	N = 93	NA	NA	NA	NA
Pyramidal (N = 162)	Measured by MEP and/or NCS							
Yes (N = 35, 19.0%_Total,_ 21.6%_FSS_)	N = 2	N = 16	N = 7	N = 17	NA	NA	NA	NA
No (N = 127, 69.0%_Total,_ 78.4%_FSS_)	N = 21	N = 79	N = 36	N = 64	NA	NA	NA	NA
Cerebellar (N = 162)	Measured by NCS							
Yes (N = 17, 9.2%_Total,_ 10.5%_FSS_)	NA	NA	N = 6	N = 4	NA	NA	NA	NA
No (N = 145, 78.8%_Total,_ 89.5%_FSS_)	NA	NA	N = 75	N = 38	NA	NA	NA	NA
Sensory (N = 162)^g^	Measured by multi-channel SSEP and/or NCS							
Yes (N = 18, 9.8%_Total,_ 11.1%_FSS_)	N = 1	N = 15	N = 5	N = 11	NA	NA	NA	NA
No (N = 144, 78.3%_Total,_ 88.9%_FSS_)	N = 6	N = 97	N = 20	N = 83	NA	NA	NA	NA
Cerebral (N = 161)	Measured by SDMT and/or MoCA							
Yes (N = 2, 1.1%_Total,_ 1.2%_FSS_)	NA	NA	NA	NA	NA	NA	N = 1	N = 1
No (N = 159, 86.4%_Total,_ 98.8%_FSS_)	NA	NA	NA	NA	NA	NA	N = 86	N = 56
Ambulation (N = 158)	Measured by NCS, MEP, multi-channel SSEP, SSR, BR							
Yes (N = 3, 1.6%_Total,_ 1.9%_FSS_)	N = 0	N = 3	N = 2	N = 1	N = 0	N = 1	NA	NA
No (N = 155, 84.2%_Total,_ 98.1%_FSS_)	N = 36	N = 83	N = 75	N = 43	N = 19	N = 81	NA	NA

Prevalence rates are displayed for the total sample and for the subgroup of patients that received the corresponding assessment(s) (indicated by subscripted ‘CNS’, ‘PNS’, ‘ANS’, ‘Cog’, ‘FSS’).

ICU, Intensive care unit; BR, Blink reflex; MEP, Motor evoked potentials; SSEP, Somatosensory evoked potentials; NCS, Nerve conduction studies, SSR, Sympathetic skin response; SDMT, Symbol digit modalities test; MoCA, Montreal Cognitive Assessment; NA, Not applicable; FSS, Functional system score; CNS,  Central nervous system; PNS,  Peripheral nervous system; ANS, Autonomous nervous system.

^a^Missing as follows: 22 neurological examination, 20 FSS Brainstem, 22 FSS Pyramidal, 22 FSS Cerebellar, 22 FSS Sensory, 23 FSS Cerebral, 26 Ambulation.

^b^Electrophysiological assessment took place in N = 139 and neuropsychological assessment took place in N = 155. N = 3 presented with abnormalities in the electrophysiological assessment that could not be classified as central, peripheral or autonomous.

^c^At the time of examination.

^d^Defined as a FSS > 0.

^e^Defined as an Expanded Disability Status Scale > 0 (visual acuity and bowel and bladder function were not taken into account because it was not possible to differentiate between new and preexisting symptoms).

^f^N = 2 presented with abnormalities in the BR that could not be unambiguously classified as peripheral or central. One of them showed no clinical signs of brainstem dysfunction in the neurological exam. The other was not examined neurologically.

^g^N = 1 with missing neurological examination presented with abnormalities in the SSEP that could not be unambiguously classified as peripheral or central.

CNS pathology occurred less frequently but was still highly prevalent, affecting 33% of patients undergoing CNS assessment. Including patients who did not receive CNS testing, the prevalence rate was 25%.

ANS pathology occurred least frequently, affecting 25% of those electrophysiologically examined and 16% of the total sample.

Eight patients were comatose, impeding most neurological investigations. In the remaining patients, brainstem and pyramidal functions were most frequently affected. Half of those patients undergoing neurological and electrophysiological assessment presented with subclinical manifestations, which were detectable in the EA but not by the neurological examination (EDSS < 1). Further, most of the clinical symptoms detected in the neurological examination did not have a corresponding finding in the EA. This was also true investigating the single FS separately, with exception of the tests for coordination of the cerebellar FS, in which most clinical symptoms were associated with pathology in the NCS.

**Figure 2:**
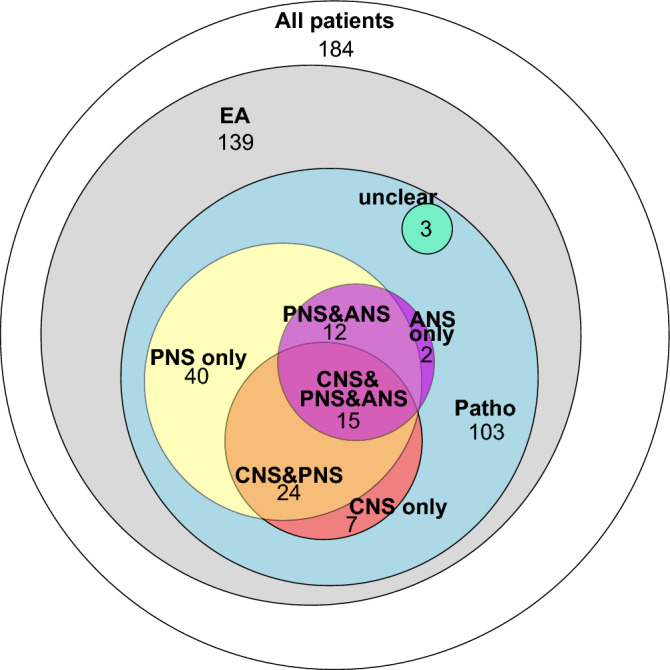
Frequencies of different nervous system affections in relation to each other, conducted electrophysiological assessments, and enrolled patients. This figure illustrates the results of the electrophysiological assessment. It shows the number of patients with pathological findings in the central, peripheral and autonomous nervous system in relation to each other, the number of conducted electrophysiological assessments, and the number of enrolled participants. The sizes of the ellipses are proportional to the number of affected participants and were estimated by the R package ‘eulerr’^42^. EA, Electrophysiological assessment; PNS, Peripheral nervous system; ANS, Autonomous nervous system; Patho, Any pathology in the EA.

Figure 2 illustrates the number of pathological findings in the different EA in relation to the number of enrolled participants and conducted EAs. It shows that EA revealed pathological findings in most patients and that most affections of the CNS and ANS were accompanied by PNS affection.

### Prevalence rates of abnormalities in the neuropsychological assessments

53% of the total sample and 63% of those undergoing cognitive assessment presented with impairment in the SDMT, MoCA or both (Table 2). No delirium was detected. Of those presenting with cognitive deficits, 36% were impaired in both tests, whereas 35% and 29% showed signs of impairment exclusively in the MoCA and SDMT, respectively.

### Association between electrophysiological readouts and death

Among patients who underwent EA of the ANS, 55% of the deceased patients had pathological findings, compared to 28% of those surviving. Independent of the outcome, peripheral pathology occurred in most patients who underwent EA with slightly more affections in the group that passed away (75%_Death_, 65%_Survival_). CNS affections were equally distributed in 50% of the deceased and 46% of the surviving patients.

Separately analyzing the single assessments, the BR revealed pathological findings in the majority of deceased patients (67%), compared to 14% in survivors. The SSEP revealed a similar pattern with 75% and 31% pathological findings in the deceased and surviving group, respectively. NCS revealed high rates of affection in both groups (90%_Death_, 67%_Survival_) whereas the MEP was less frequently abnormal (57%_Death_, 32%_Survival_). Absolute frequencies of pathologies in the EA for both groups are presented in Supplementary Figure S2.

**Figure 3:**
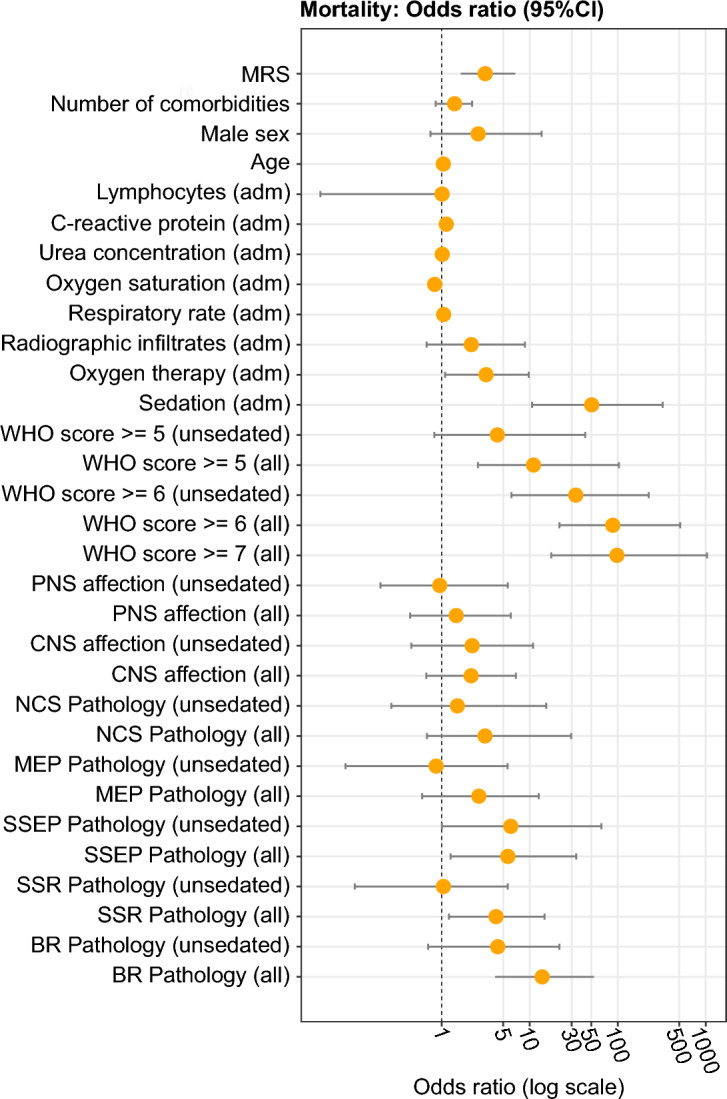
Odds ratio of mortality. This figure shows the odds ratios of mortality (with 95% confidence interval) on the log scale. All scores refer to the time of examination if not indicated otherwise. Number of comorbidities were defined according to the extended Charlson comorbidity index^26^. No results for the Glasgow coma scale^19^ at admission are displayed because all patients achieved the highest possible score. See Table 3 for detailed statistics. CI, Confidence interval; adm, Score refers to the time of admission to the hospital; MRS, Modified Rankin Scale; WHO score, WHO clinical progression scale; PNS, Peripheral nervous system; CNS, Central nervous system; NCS, Nerve conduction studies; MEP, Motor evoked potentials; SSEP, Somatosensory evoked potentials; SSR, Sympathetic skin response; BR, Blink reflex.

Logistic regression revealed independent associations of abnormalities in the BR, SSR, and SSEP with mortality. Odds ratios are presented in Fig. 3 and Table 3. Investigating the clinical status, sedation, and WHO scores ≥ 7 and ≥ 6 were strongly associated with death. Sedation is known to influence BR and SSR but not SSEP and, in line with this, the association with mortality lost significance for the BR and SSR when sedated patients were excluded, but remained significant for the SSEP. After including age and sex as covariates, no effects remained significant in unsedated patients (Supplementary Table 6).

**Table 3:** Odds ratios of mortality (with 95% confidence interval).

Predictor	95% CI			
	OR	Lower	Upper	*p*-value
MRS	3.13	1.66	6.88	**0.00058**
Number of comorbidities	1.40	0.86	2.22	0.17
Male sex	2.60	0.75	13.63	0.14
Age	1.04	1.01	1.09	**0.012**
Lymphocytes (adm)	1.01	0.04	1.18	0.93
C-reactive protein (adm)	1.13	1.06	1.18	**0.00014**
Urea concentration (adm)	1.02	1.00	1.03	**0.0053**
Oxygen saturation (adm)	0.83	0.74	0.91	** < 0.0001**
Respiratory rate (adm)	1.05	0.94	1.16	0.34
Radiographic infiltrates (adm)	2.17	0.68	8.84	0.20
Oxygen therapy (adm)	3.20	1.09	9.76	**0.034**^**#**^
Sedation	50.60	10.65	324.94	** < 0.0001**
WHO score ≥ 5 (unsedated)	4.29	0.82	42.73	0.086
WHO score ≥ 5 (all)	11.05	2.58	103.09	**0.00044**
WHO score ≥ 6 (unsedated)	33.37	6.22	225.40	** < 0.0001**
WHO score ≥ 6	87.94	21.75	513.58	** < 0.0001**
WHO score ≥ 7 (all)	97.93	17.57	1034.42	** < 0.0001**
PNS affection (unsedated)	0.95	0.20	5.63	0.95
PNS affection (all)	1.46	0.44	6.10	0.55
CNS affection (unsedated)	2.22	0.45	10.92	0.31
CNS affection (all)	2.16	0.67	6.98	0.19
NCS pathology (unsedated)	1.51	0.27	15.42	0.66
NCS pathology (all)	3.11	0.68	29.69	0.14
MEP pathology (unsedated)	0.87	0.08	5.61	0.89
MEP pathology (all)	2.56	0.60	12.66	0.19
SSEP pathology (unsedated)	6.10	1.01	65.13	**0.049**^**#**^
SSEP pathology (all)	5.65	1.26	33.86	**0.023**^**#**^
SSR pathology (unsedated)	1.05	0.10	5.64	0.96
SSR pathology (all)	4.15	1.21	14.75	**0.024**
BR pathology (unsedated)	4.34	0.70	21.78	0.11
BR pathology (all)	13.87	4.06	53.82	** < 0.0001**

All scores refer to the time of examination if not indicated otherwise. Number of comorbidities were defined according to the extended Charlson comorbidity index^26^. No results for the Glasgow coma scale^19^ at admission are displayed because all patients achieved the highest possible score. *p*-values < 0.05 are in boldface. ^#^indicates that significance was lost after controlling for age and sex.

CI, Confidence interval; adm, Score refers to the time of admission to the hospital; MRS, Modified Rankin Scale; WHO score, WHO clinical progression scale; PNS, Peripheral nervous system; CNS, Central nervous system; NCS, Nerve conduction studies; MEP, Motor evoked potentials; SSEP, Somatosensory evoked potentials; SSR, Sympathetic skin response; BR, Blink reflex.

Analysis of the raw data revealed significant associations between death and the N20 (mean of both sides of the body) as well as iR2 (left and right) not only in the total sample but also in the subsample of unsedated patients (Supplementary Figure S3). Excluding patients with a history of DM (*n* = 39) resulted in a subsample of only six deceased patients and associations of abnormalities in the EA with mortality lost significance (Supplementary Figure S4). However, the N20 and iR2 were still associated with death (Supplementary Figure S5).

Among the parameters already described to be predictive of the patient’s outcome^26,27^, significant associations with mortality were confirmed for oxygen saturation and need of oxygen therapy, C-Reactive protein, and age. However, the association of need of oxygen therapy lost significance after controlling for age and sex.

The Kaplan–Meier-Curves, Hazard Ratios and the number of remaining patients under observation for all EAs from the time of assessment until discharge are presented in Supplementary Figure S6, illustrating that death mostly occurred within 30 days of hospitalization.

### Time-trend of pathologies

**Figure 4:**
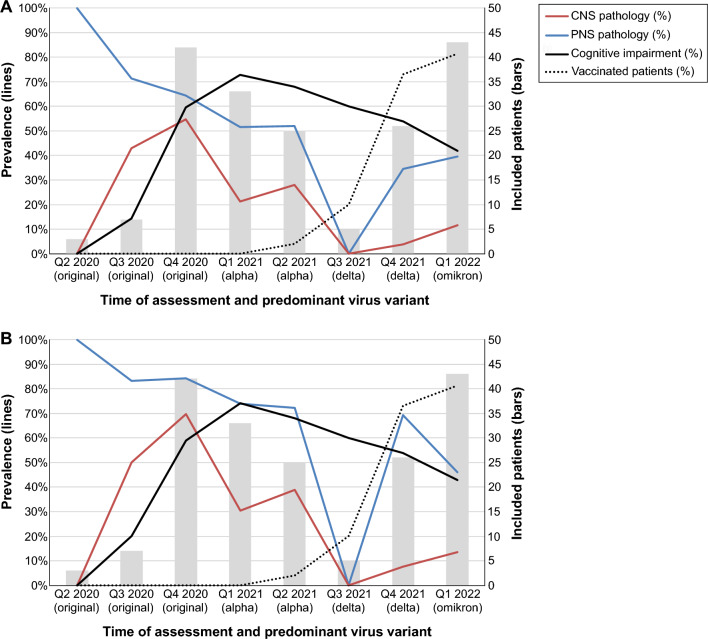
Time-trend of neurological manifestations. This figure shows the prevalence rates of central nervous system affection (red), peripheral nervous system affection (blue) and cognitive impairment (black) over time. The rate of vaccinated patients (dotted) and the predominant virus variant for each period are presented as well. Part A displays the prevalence rates based on the number of all included patients (*N* = 184). Part B displays the prevalence rates based only on the number of patients receiving the corresponding assessment(s). E.g., prevalence of cognitive impairment is based only on patients participating in neuropsychological assessment (*N* = 157). Please refer to Table 2 for overall prevalence rates and number of assessed patients. PNS, Peripheral nervous system; CNS, Central nervous system.

Due to the limited sample size, it was impossible to analyze the effects of factors such as virus variant, immunization status and treatment option on the presence of neurological affections. Although we could not statistically control for these factors during our analyses, the number of patients with CNS, PNS and cognitive affections in relation to the number of included patients and the number of vaccinated patients are presented in Fig. 4.

## Discussion

We extensively investigated neurological function and cognitive performance in a large population of hospitalized patients with moderate to severe COVID-19 and report high prevalence rates of CNS and especially PNS affections during the acute phase of the infection in this cohort. In line with previous studies^28^, we also report high rates of cognitive impairment. More than half of the patients presented with cognitive impairment, which was evitable in below average performance in the MoCA and/or reduced IPS.

We present a detailed neurological profile based on objective and standardized assessments, classifying not only clinical symptoms but also subclinical pathologies. Further, we provide information to help identify patients who may be at greater risk for complications and may benefit from more aggressive and earlier treatment. Not only high WHO scores^18^, reflecting respiratory failure, are associated with an increased risk of mortality but also SSEP pathology was identified as an independent risk factor. Although this effect lost statistical significance after controlling for age and sex, this could be of high value for patient care and possibly even of psychosocial and economic relevance.

In contrast to previous results^29^, PNS affections were more common than CNS affections. However, our estimates may be biased by patients with previously undiagnosed polyneuropathy (PNP), e.g. due to DM, alcohol consumption, malnutrition or other risk factors of PNP, as we only excluded patients with a known history of PNP. Importantly, prevalence of PNS affection remained stable when excluding patients with DM, suggesting that PNS pathology may indeed occur as a consequence of COVID-19. Our results are also in accordance with previous studies reporting high prevalence rates of (sub)clincal PNS affections, even in patients with mild COVID-19^30^ and the subacute state^31^. However, most previous studies did not exclude patients with neurological conditions prior to COVID-19, thereby potentially overestimating PNS pathologies. Our study on patients without previous neurological affections, therefore adds important value to the current literature on neurological affections in patients with COVID-19.

The major limitation of our study is the lack of a control group. Our study was designed to include hospitalized patients with pneumonias of other origins as a control group. However, in the context of diverse measures to prevent the spread of SARS-CoV-2, the incidence of other pneumonias was very low and due to the exclusion criterion of confounding neurological disorders we could only include 13 of 91 approached control patients. Precluding meaningful comparison, we chose not to include these patients in the analyses. Thus, the reported affections cannot be considered specific for COVID-19 and need to be interpreted in the context of other factors associated with neurological manifestations, such as ICU treatment. ICU treatment is well known to be associated with neurological manifestations such as critical illness polyneuropathy or myopathy (CIP/CIM), but prevalence rates are strongly influenced by the studied population, time of assessment, risk factors and diagnostic criteria^32^. Thus, comparison with the prevalence rates found in our cohort of COVID-19 patients is difficult.

In patients with acute respiratory distress syndrome (ARDS), prevalence of ICU-acquired weakness, summarizing both CIP and CIM, ranges from 36% at hospital discharge to 60% following sedation^33,34^. Importantly, prevalence of PNS pathology in our subgroup of ICU patients exceeds the highest rate in ARDS (85.7% considering all ICU patients/ 90% considering all ICU patients that underwent PNS assessment). Further, the majority of our participants was not treated in the ICU and median time of hospitalization was rather short (8 days). In line with this, a study comparing clinical and electrophysiological data of patients with ICU-acquired weakness between patients with COVID-19 and other diseases reported significantly more CIP in the COVID-19 cohort^35^. Based on findings of a retrospective cohort study comparing neurological and psychiatric manifestations after COVID-19 and other respiratory tract infections, we further believe that our reported rates are higher than what could be expected from other infections^36^. Nonetheless, studies comparing neurological manifestations in hospitalized COVID-19 patients with hospitalized SARS-CoV-2 negative patients are urgently needed and already underway.

At the time of study initiation, evidence regarding neurological manifestations in patients with COVID-19 was scarce and limited to case series, prohibiting power-calculation. Therefore, our study has to remain descriptive in nature and deductions are limited to our study population and center. However, our center is representative of hospitals treating patients with COVID-19 in Germany.

Patients were recruited regardless of subjective (neurological) symptoms. It’s possible that those with subjective neurological complaints were more inclined to participate in our study, potentially inflating the prevalence rates of neurological affections. Unfortunately, selection bias is a well-known limitation of clinical research due to the voluntary nature of study participation. To avoid further bias in our prevalence rates, we do not only report prevalence rates based on the number of patients who received assessment of CNS/PNS function but also based on the number of all included patients. While the former may overestimate the ‘true’ rate, the latter is rather conservative and probably an underestimation. We, therefore, believe that the ‘true’ rate falls between the two estimates.

Further limitations are the lack of neuro(psycho)logical or even electrophysiological evaluation prior to the infection and the lack of ambulatory patients, who could not be investigated due to quarantine measures. Additionally, neuropsychological testing may have been influenced by factors, such as anxiety and depression, potentially linked to the pandemic in general but also to the hospitalization with COVID-19. Indeed, there had been a small increase in mental health symptoms during the first months of the pandemic, which did, however, decline over time^37^. Looking at the time-trend, rates of cognitive impairment remained high throughout the course of the pandemic despite immunization, more treatment options, less fatal virus variants, and decreasing levels of mental health symptoms. This suggests, that cognitive impairment occurred independent of these factors in our cohort. However, cross-sectional neuropsychological assessments can only inform about the cognitive performance in a specific situation and timepoint, while longitudinal assessments are needed to inform about the persistence of deficits. Indeed, longitudinal studies have shown, that cognitive deficits may persist up to one year after the acute infection^38–40^.

Confounding factors such as immunization status and variable treatment may have influenced our predictive measures. Importantly, however, we controlled for the most important confounding factor, namely preexisting neurological conditions, by strictly excluding patients with a previous history of neurological disorders. Even though this criterion drastically reduced our final cohort, this was the best procedure to prohibit further selection bias and to ensure that neurological affections detected in this study were unlikely present prior to COVID-19. Exploratory, we also controlled for age and sex in our predictive analyses. As expected, based on the well-known influence of age and sex, including these factors, dramatically decreased the predictive value of the neurological affections.

It would be interesting to compare the prevalence rate and prognostic value of the reported affections for different SARS-CoV-2 variants. The severity rates have decreased over time as a result of mutations of the virus, vaccinations and novel treatment options. With the limited number of cases of each variant in this study, it was impossible to control for these strong confounding factors to compare the effect of different SARS-CoV-2 variants, immunization status and treatment. However, we provide a graphical illustration of the number of patients with CNS, PNS and cognitive affections in relation to the number of included patients and the number of vaccinated patients. Although entirely descriptive, it suggests that neurological affections occurred less frequently during the course of the pandemic. Larger studies are needed to investigate the relevance of the virus variant, immunization status, treatment, and other factors.

Future studies should also include neuroimaging data to increase sensitivity of detecting possible CNS affections that may not be revealed by electrophysiological and neurological examination. Further, the prognostic relevance of sensitive laboratory measures of brain injury (e.g. blood neurofilament light chain protein (NfL) as a marker of neuroaxonal damage and nervous system involvement)^41^ should be taken into account. In a recent IPD meta-analysis, NfL levels were not only elevated in hospitalized COVID-19 patients without major CNS manifestations but also associated with poor clinical outcomes^3^. Although further investigations are warranted, these results indicate that overt neurological symptoms may merely represent the surface manifestations of a more complex and multifaceted underlying issue.

In conclusion, we report high rates of CNS and PNS affection in a cohort of hospitalized patients with moderate to severe COVID-19 during the acute infection, objectively assessed by electrophysiological examination. While somatosensory affections may have an independent prognostic value for mortality in the acute phase, affections of the PNS seem to occur very frequently. EA, notably SSEP, may be helpful in the clinical routine to identify patients at high risk for unfavorable outcomes and to allocate limited resources for the management of post-COVID-19 sequelae.

### Supplementary Information

Supplementary Information.

## Data Availability

Deidentified participant data, a data dictionary, and informed consent forms (German only) will be made available on request by researchers working in related fields three months after publication. Data will be provided by the corresponding author.
