# Supplementary material for: Prevalence and prognostic value of neurological affections in hospitalized patients with moderate to severe COVID-19 based on objective assessments
Source: Sci Rep. 2023 Nov 10;13:19619. doi: 10.1038/s41598-023-46124-w (PMC10638293; doi:10.1038/s41598-023-46124-w)
Supplement: Supplementary file 1 — Supplementary Information. [file 41598_2023_46124_MOESM1_ESM.pdf]

Prevalence and prognostic value of neurological affections in hospitalized patients with moderate to severe COVID-19 based on objective assessments

Supplementary Material

Table of content

1. Supplementary Methods 2
   Implemented disability scores 2
   Calculation of neuropsychological z-scores 2
2. Supplementary Tables 4
   Supplementary Table S1 Normal reference range of the parameters of interest in the nerve conduction studies 4
   Supplementary Table S2 Normal reference range of the parameters of interest in the sympathetic skin response 4
   Supplementary Table S3 Normal reference range of the parameters of interest in the blink reflex 4
   Supplementary Table S4 Normal reference range of the parameters of interest in the motor evoked potentials 4
   Supplementary Table S5 Normal reference range of the parameters of interest in the somato-sensory evoked potentials 5
   Supplementary Table S6 Odds ratios of mortality after controlling for age and sex (with 95% confidence interval) 6
3. Supplementary Figures 7
   Supplementary Figure S1 Individual data for each assessment and timepoint 7
   Supplementary Figure S2 Frequencies of pathologies in the electrophysiological assessment in patients who died versus survived 11
   Supplementary Figure S3 Odds Ratios (95% CI) of mortality based on the raw values in the electrophysiological assessment 13
   Supplementary Figure S4 Odds Ratios (95% CI) of mortality excluding patients with diabetes mellitus 15
   Supplementary Figure S5 Odds Ratios (95% CI) of mortality based on the raw values in the electrophysiological assessment excluding patients with diabetes mellitus 17
   Supplementary Figure S6 Kaplan-Meier-Curves and Cox proportional hazard ratios for each electrophysiological assessment including and excluding sedated patients 19
4. References 22

Supplementary Methods

Implemented disability scores

The Expanded Disability Status Scale (EDSS)[1] is a widely accepted method to quantify disability related to Multiple Sclerosis on an ordinal scale ranging from 0 (no neurological signs) to 10 (death due to Multiple Sclerosis). It's calculated by separate evaluation of the following seven Functional Systems (FS) and ambulation: visual, brainstem, pyramidal, cerebral, cerebellar, sensory, and bowel and bladder. Each of the FS is scored on an ordinal clinical rating scale from 0 (no signs) to 5 or 6 (depending on the FS), resulting in a FS score (FSS). Only signs which have not already been present prior to the infection were taken into account. Because the differentiation between new and preexisting symptoms was not unequivocally possible for the visual and bowel and bladder FS, these two scores were not included in the present study. If a single or a combination of FS was not assessable (e.g., ambulation in bedridden patients), all other FS were assessed.

The Modified Rankin Scale[2] is the most widely used clinical outcome measure for stroke patients, measuring the degree of disability after a stroke on an ordinal scale from 0 (no symptoms) to 6 (death). We adapted this score to COVID-19 symptoms. The INCAT disability score[3] measures activity limitation due to disability of the upper and lower limbs and is primarily used in research on inflammatory polyneuropathy. Arm and leg disability are both classified from 0 (no symptoms) to 5. The Barthel Index[4] completed our test battery as an ordinal measure of performance in ten activities of daily living and mobility.

Calculation of neuropsychological z-scores

In the German norms of the Symbol Digit Modalities Test (SDMT)[5], the total number of correct digits is transformed into a z-score correcting for age and education. This correction is applied by using a formula of the following format:

*SDMT z-score = a * (total number of correct items) - b + c * (age category) - d * (education category)*

There are four age (1 = 0-29 years, 2 = 30-39 years, 3 = 40-49 years, 4 = 50-59 years) and three education groups (0 = no formal school diploma or lowest secondary school diploma (Hauptschule; ≤ 9 years), 1=intermediate secondary school diploma (Mittlere Reife, 10-11 years), 2 = highest secondary school diploma (Fachabitur/Abitur, 12-13 years, regional differences)).

Unfortunately, almost half of our participating patients (N=64, 41%) fell outside the range of the highest age category. Subjects older than 59 years old were not considered in the development of this normative formula and therefore, they cannot be assigned to any of the available age groups. To

avoid overestimation of cognitive impairment in elderly patients by assigning z-scores based on the highest available age category (50-59 years), we added two categories, inspired by the range of those provided in the German norms: 5=60-69 years, 6=≥70 years. Following the published cut-off-score for the other age groups, slowed information processing speed was defined based on a z-score of ≤ -1.68. To explore the effect of this adaptation, we analyzed the number of patients classified as impaired using 1) our adapted age categories and 2) four age categories only, assigning all patients older than 50 years to the same category. Six patients were classified as impaired in 2) but not in 1). Total scores of the Montreal Cognitive Assessment (MoCA, version 7)[6] were transformed into z-scores based on German norms, correcting for sex, age, and education.[7] In line with common research practice,[8] patients with z-scores < -1.645 were classified as cognitively impaired.

Supplementary Tables

Supplementary Table S1 Normal reference range of the parameters of interest in the nerve conduction studies

Nerve	Distal latency (ms)	Amplitude (mV)	Conduction velocity (m/s)	F-wave latency (ms)
Motor nerves				
Tibialis	<5.8	>5	>40	<58
Ulnaris	<3.4	>5	>45	<32
Sensory nerves				
Suralis	-	>5	>40	-
Ulnaris	-	>5	>45	-

Note: Obtained by assessments of representative healthy controls defining cut-off values >2.5 standard deviations from the means of the controls.

Supplementary Table S2 Normal reference range of the parameters of interest in the sympathetic skin response

Extremity	Distal latency (ms)	Amplitude (mV)
Arms	<1.63	>260
Legs	<2.44	>240

Note: Obtained by assessments of representative healthy controls defining cut-off values >2.5 standard deviations from the means of the controls.

Supplementary Table S3 Normal reference range of the parameters of interest in the blink reflex

R1 latency (ms)	Ipsilateral R2 latency (ms)	Contralateral R2 latency (ms)	Side-to-side difference
<12	<40	<40	<4.3

Note: Obtained by assessments of representative healthy controls defining cut-off values >2.5 standard deviations from the means of the controls.

Supplementary Table S4 Normal reference range of the parameters of interest in the motor evoked potentials

Nerve	Age (years)	Cortical latency (ms)	Central motor latency (ms)
Medianus	18-29	≤24.2	≤7.8
	30-59	≤23.4	≤7.8
	>60	≤24.5	≤8.7
Tibialis	18-29	≤33.4	≤17.2
	30-59	≤35.7	≤17.7
	>60	≤36.1	≤19.9

Note: Obtained by assessments of representative healthy controls defining cut-off values >2.5 standard deviations from the means of the controls.

Supplementary Table S5 Normal reference range of the parameters of interest in the somato-sensory evoked potentials

Height (cm)	P40 (ms)	N20 (ms)
≤150	38.6	19.3
151	38.8	19.4
152	39.1	19.5
153	39.3	19.7
154	39.6	19.8
155	39.8	19.9
156	40.1	20.05
157	40.3	20.15
158	40.6	20.3
159	40.9	20.45
160	41.1	20.5
161	41.4	20.7
162	41.6	20.8
163	41.9	20.9
164	42.1	21.05
165	42.4	21.2
166	42.7	21.34
167	42.9	21.4
168	43.2	21.6
169	43.4	21.7
170	43.7	21.85
171	43.9	21.95
172	44.2	22.1
173	44.5	22.25
174	44.7	22.3
175	45.0	22.5
176	45.2	22.6
177	45.5	22.7
178	45.7	22.9
179	46.0	23.0
180	46.3	23.15
181	46.5	23.25
182	46.8	23.4
183	47.0	23.5
184	47.3	23.6
185	47.5	23.8
186	47.8	23.9
187	48.1	24.05
188	48.3	24.15
189	48.6	24.3
190	48.8	24.4
≥191	49.1	24.55

Note: Obtained by assessments of representative healthy controls defining cut-off values >2.5 standard deviations from the means of the controls.

Supplementary Table S6 Odds ratios of mortality after controlling for age and sex (with 95% confidence interval)

		95% CI		
**Predictor**	OR	Lower	Upper	*p-value*
MRS	3.02	1.60	6.72	**0.00083**
Number of comorbidities	1.16	0.66	1.92	0.06
Male sex	2.28	0.64	12.08	0.22
Age	1.04	1.01	1.09	**0.017**
Lymphocytes (adm)	0.97	0.04	1.14	0.77
C-reactive protein (adm)	1.11	1.05	1.20	**0.00058**
Urea concentration (adm)	1.01	1.00	1.02	**0.033**
Oxygen saturation (adm)	0.84	0.74	0.92	**0.0001**
Respiratory rate (adm)	1.04	0.92	1.14	0.53
Radiographic infiltrates (adm)	1.96	0.58	8.30	0.29
Oxygen therapy (adm)	3.01	0.999	9.46	0.05
Sedation	43.62	8.86	305.16	**<0.0001**
WHO score ≥ 5 (unsedated)	4.20	0.80	41.93	0.09
WHO score ≥ 5 (all)	10.41	2.40	97.89	**0.0008**
WHO score ≥ 6 (unsedated)	26.99	4.99	183.30	**0.0002**
WHO score ≥ 6	74.07	17.97	447.75	**<0.0001**
WHO score ≥ 7 (all)	140.20	19.61	2639.00	**<0.0001**
PNS affection (unsedated)	0.64	0.13	3.89	0.60
PNS affection (all)	1.00	0.28	4.26	>0.99
CNS affection (unsedated)	2.03	0.41	10.23	0.37
CNS affection (all)	1.97	0.60	6.52	0.26
NCS Pathology (unsedated)	0.99	0.16	10.52	0.99
NCS Pathology (all)	2.23	0.46	21.77	0.35
MEP Pathology (unsedated)	0.71	0.07	4.51	0.73
MEP Pathology (all)	2.24	0.51	10.74	0.28
SSEP Pathology (unsedated)	4.40	0.68	48.87	0.12
SSEP Pathology (all)	4.15	0.86	25.94	0.08
SSR Pathology (unsedated)	0.85	0.08	4.68	0.86
SSR Pathology (all)	3.59	1.03	12.95	**0.04**
BR Pathology (unsedated)	3.31	0.53	16.79	0.18
BR Pathology (all)	11.81	3.39	46.87	**0.0001**

Note. All scores refer to the time of examination if not indicated otherwise. Number of comorbidities were defined according to the extended Charlson comorbidity index.[9] No results for the Glasgow coma scale [10] at admission are displayed because all patients achieved the highest possible score. p-values <0.05 are in boldface.

CI = confidence interval; adm= score refers to the time of admission to the hospital; MRS = Modified Rankin Scale; WHO score = WHO clinical progression scale; PNS = peripheral nervous system; CNS = central nervous system; NCS = nerve conduction studies; MEP = motor evoked potentials; SSEP = somatosensory evoked potentials; SSR = sympathetic skin response; BR = blink reflex.

Supplementary Figures

Supplementary Figure S1 Individual data for each assessment and timepoint

This figure shows all raw data from each assessment and timepoint per patient. Data from the same patient are connected. Information regarding the hospital outcome and pathology of the assessment are incorporated. Part A-D show all relevant parameters of the MEP. Part E&F show all relevant parameters of the SSEP. Part G&H show all relevant parameters of the BR. Part I-L show all relevant parameters of the NCS. Part M&N show all relevant parameters of the SSR. Part O&P show all relevant parameters of the neuropsychological assessment.

sCV = sensory nerve conduction velocity, SNAP = sensory nerve action potential, CMAP = compound muscle action potential, mCV = motor nerve conduction velocity, CMCT = central motor conduction time, MEP = motor evoked potentials, SSEP = somatosensory evoked potentials, NCS = nerve conduction studies, SSR = sympathetic skin response, BR = blink reflex, SDMT = Symbol Digit Modalities Test, MoCA = Montreal Cognitive Assessment.

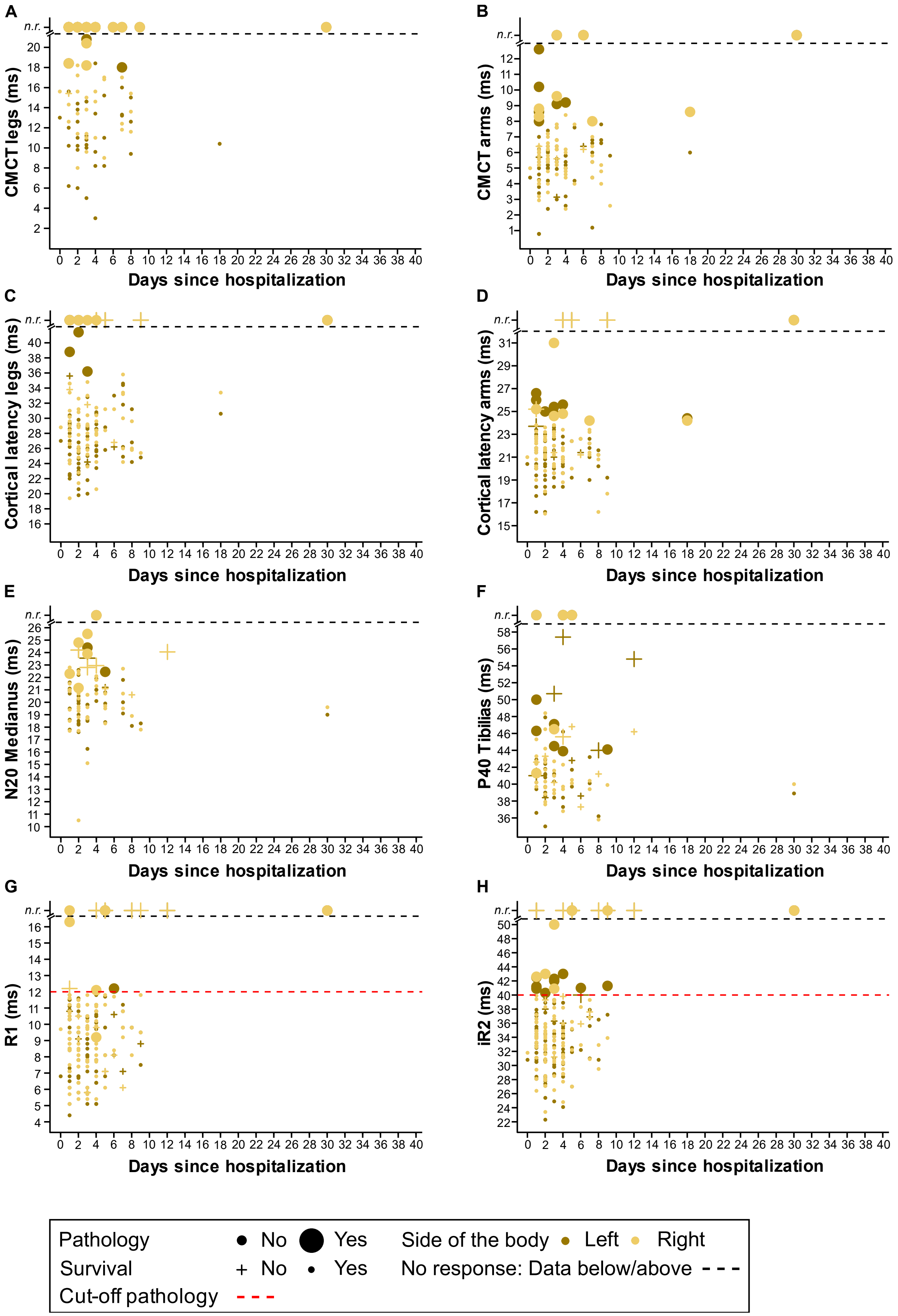
A
n.r.
CMCT legs (ms)
Days since hospitalization
B
n.r.
CMCT arms (ms)
Days since hospitalization
C
n.r.
Cortical latency legs (ms)
Days since hospitalization
D
n.r.
Cortical latency arms (ms)
Days since hospitalization
E
n.r.
N20 Medianus (ms)
Days since hospitalization
F
n.r.
P40 Tibilias (ms)
Days since hospitalization
G
n.r.
R1 (ms)
Days since hospitalization
H
n.r.
iR2 (ms)
Days since hospitalization
Pathology No Yes
Survival No Yes
Cut-off pathology
Side of the body Left Right
No response: Data below/above

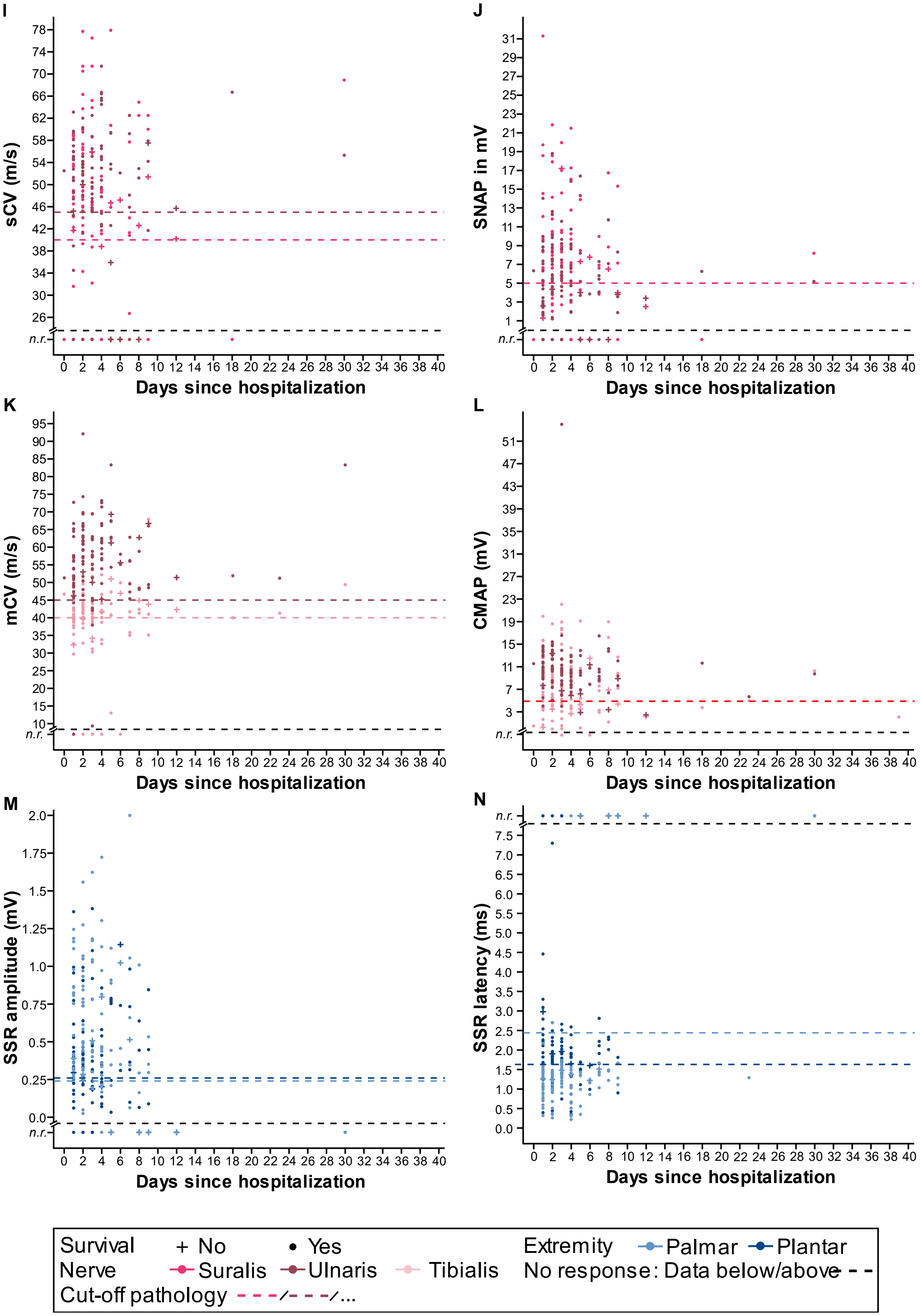
I
sCV (m/s)
Days since hospitalization
J
SNAP in mV
Days since hospitalization
K
mCV (m/s)
Days since hospitalization
L
CMAP (mV)
Days since hospitalization
M
SSR amplitude (mV)
Days since hospitalization
N
SSR latency (ms)
Days since hospitalization
n.r.
Survival + No • Yes
Nerve Suralis Ulnaris Tibialis
Cut-off pathology /...
Extremity Palmar Plantar
No response: Data below/above

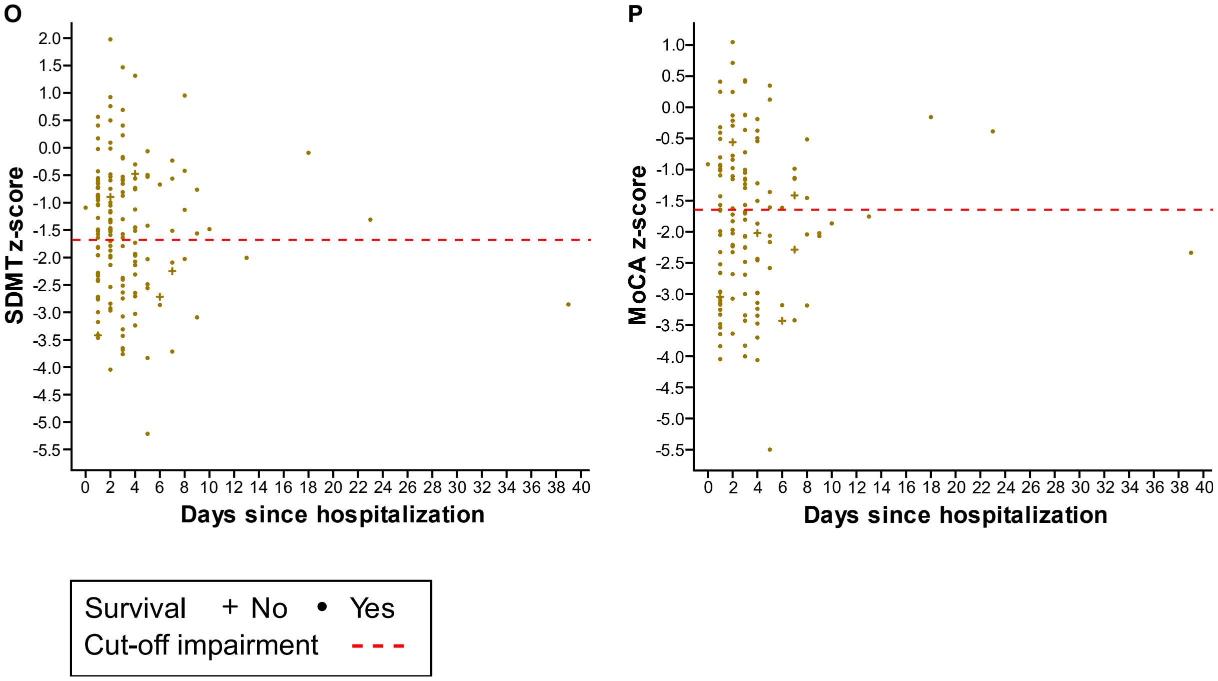
O
SDMT z-score
Days since hospitalization
P
MoCA z-score
Days since hospitalization
Survival + No • Yes
Cut-off impairment

Supplementary Figure S2 Frequencies of pathologies in the electrophysiological assessment in patients who died versus survived

This figure shows the frequencies of pathological and normal results in the electrophysiological assessment stratified by the clinical outcome (death vs. survival).

CNS = central nervous system, PNS = peripheral nervous system, ANS = autonomous nervous system, BR = blink reflex, MEP = motor evoked potentials, SSEP = somatosensory evoked potentials, NCS = nerve conduction studies, DM = diabetes mellitus.

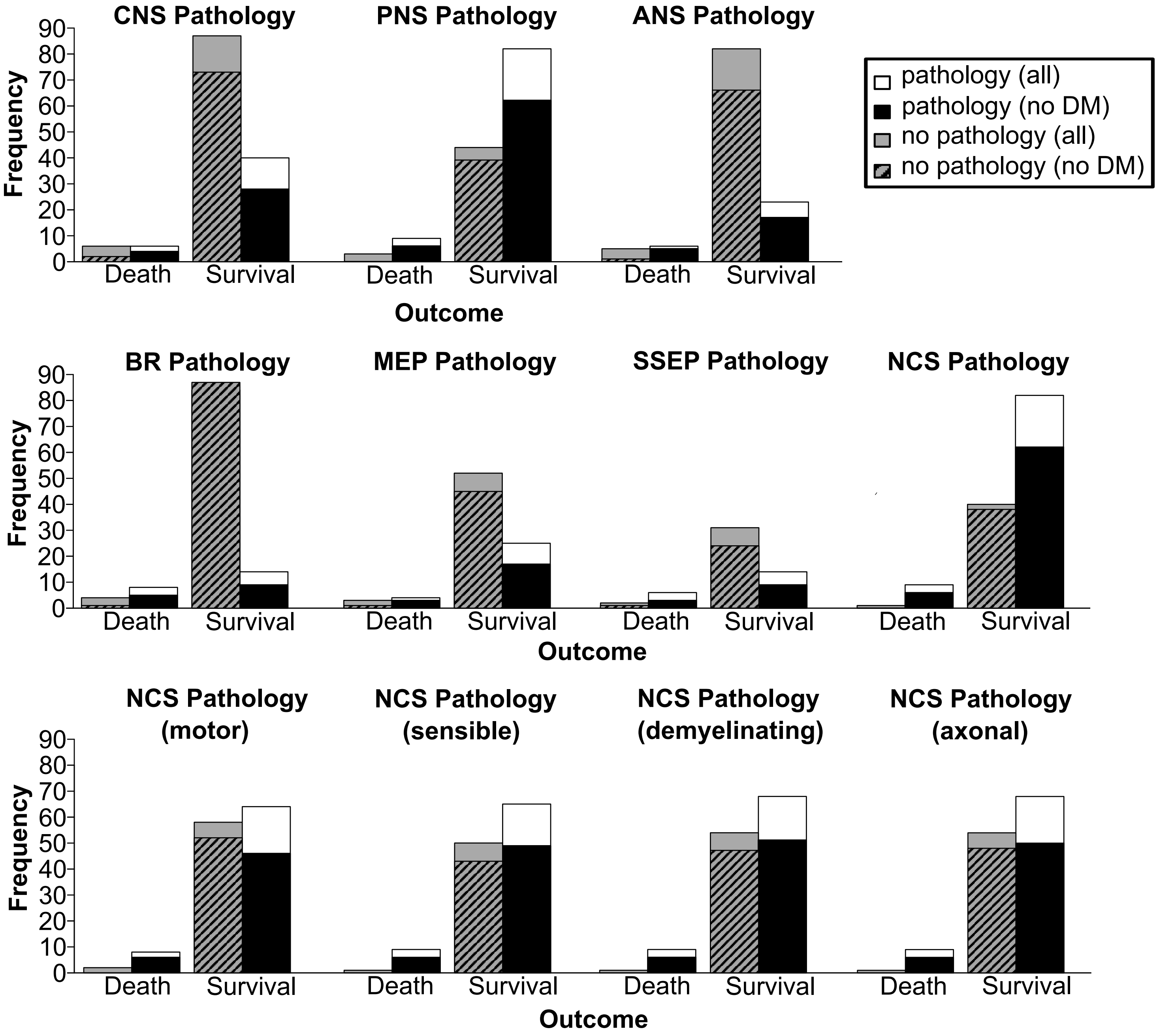
CNS Pathology
PNS Pathology
ANS Pathology
pathology (all)
pathology (no DM)
no pathology (all)
no pathology (no DM)
Frequency
0
10
20
30
40
50
60
70
80
90
Death
Survival
Outcome
BR Pathology
MEP Pathology
SSEP Pathology
NCS Pathology
NCS Pathology (motor)
NCS Pathology (sensible)
NCS Pathology (demyelinating)
NCS Pathology (axonal)

Supplementary Figure S3 Odds Ratios (95% CI) of mortality based on the raw values in the electrophysiological assessment

This figure shows the odds ratios of mortality (with 95% confidence interval) on the log scale, based on the raw values in the electrophysiological assessment. The mean of the left and right sight of the body was used for the N20, P40, CMCT and cortical latency. *p*-values < .05 are in boldface.

CI = confidence interval, sCV = sensory nerve conduction velocity, SNAP = sensory nerve action potential, CMAP = compound muscle action potential, mCV = motor nerve conduction velocity, SSR = sympathetic skin response, CMCT = central motor conduction time.

sCV Ulnaris (unsedated): 1.06 (0.99-1.13), p=.097; sCV Ulnaris: 1.05(1.00-1.10), p=.78; sCV Suralis (unsedated): 0.99 (0.94-1.04), p=.97; sCV Suralis: 0.99 (0.95-1.02), p=.53; SNAP Ulnaris (unsedated): 1.10 (0.98-1.24), p=.097; SNAP Ulnaris: 1.09 (1.00-1.18), p=.056; SNAP Suralis (unsedated): 1.00 (0.93-1.06), p=.90; SNAP Suralis: 0.99 (0.94-1.04), p=.78; CMAP Tibialis (unsedated): 0.84 (0.65-1.02), p=.087; CMAP Tibialis: 0.81 (0.66-0.96), p=**.0096**; CMAP Ulnaris (unsedated): 1.01 (0.62-1.08), p=.87; CMAP Ulnaris: 0.68 (0.52-0.85), p=**.00088**; mCV Tibialis (unsedated): 0.93 (0.83-1.03), p=.18; mCV Tibialis: 0.97 (0.89-1.03), p=.41; mCV Ulnaris (unsedatd): 0.95 (0.90-1.01), p=.11; mCV Ulnaris: 0.98 (0.94-1.04), p=.56; N20 (unsedated): 1.71 (1.08-3.19), p=**.023**; N20 (mean): 1.63 (1.12-2.66), p=**.011**; P40 (unsedated): 1.04 (0.88-1.17), p=.61; P40 (mean): 1.06 (0.93-1.18), p=.36; SSR latency (plantar, unsedated): 1.07 (0.49-1.51), p=.75; SSR latency (plantar): 1.47 (1.19-1.83), p=**.00041**; SSR latency (palmar, unsedated): 2.10 (0.38-7.71), p=.37; SSR latency (palmar): 4.82 (2.34-11.12), p**<.0001**; CMCT (arms, unsedated): 1.00 (1.00-1.01), p=.47; CMCT (arms): 1.00 (0.99-1.00), p=.56; CMCT (legs, unsedated): 1.00 (0.99-1.00), p=.85; CMCT (legs): 1.00 (0.99-1.00), p=.80; Cortical latency (arms, unsedated): 1.23 (0.70-2.02), p=.45; Cortical latency (arms): 1.01 (1.00-1.01), p=**.00028**; Cortical latency (legs, unsedated): 1.00 (0.99-1.00), p=.78; Cortical latency (legs): 1.00 (1.00-1.01), p=**.0057**; iR2 (right, unsedated): 1.13 (1.03-1.25), p=.0**15**; iR2 (right): 1.19 (1.12-1.29), p<**.0001**; iR2 (left, unsedated): 1.19 (1.06-1.36), p=**.0042**; iR2 (left): 1.24 (1.15-1.37), p<**.0001**; R1 (right, unsedated): 0.96 (0.62-1.31), p=.85; R1 (right): 1.29 (1.12-1.50), p=**.00050**; R1 (left, unsedated): 0.98 (0.64-1.49), p=.91; R1 (left): 1.39 (1.09-1.80), p=**.0089**

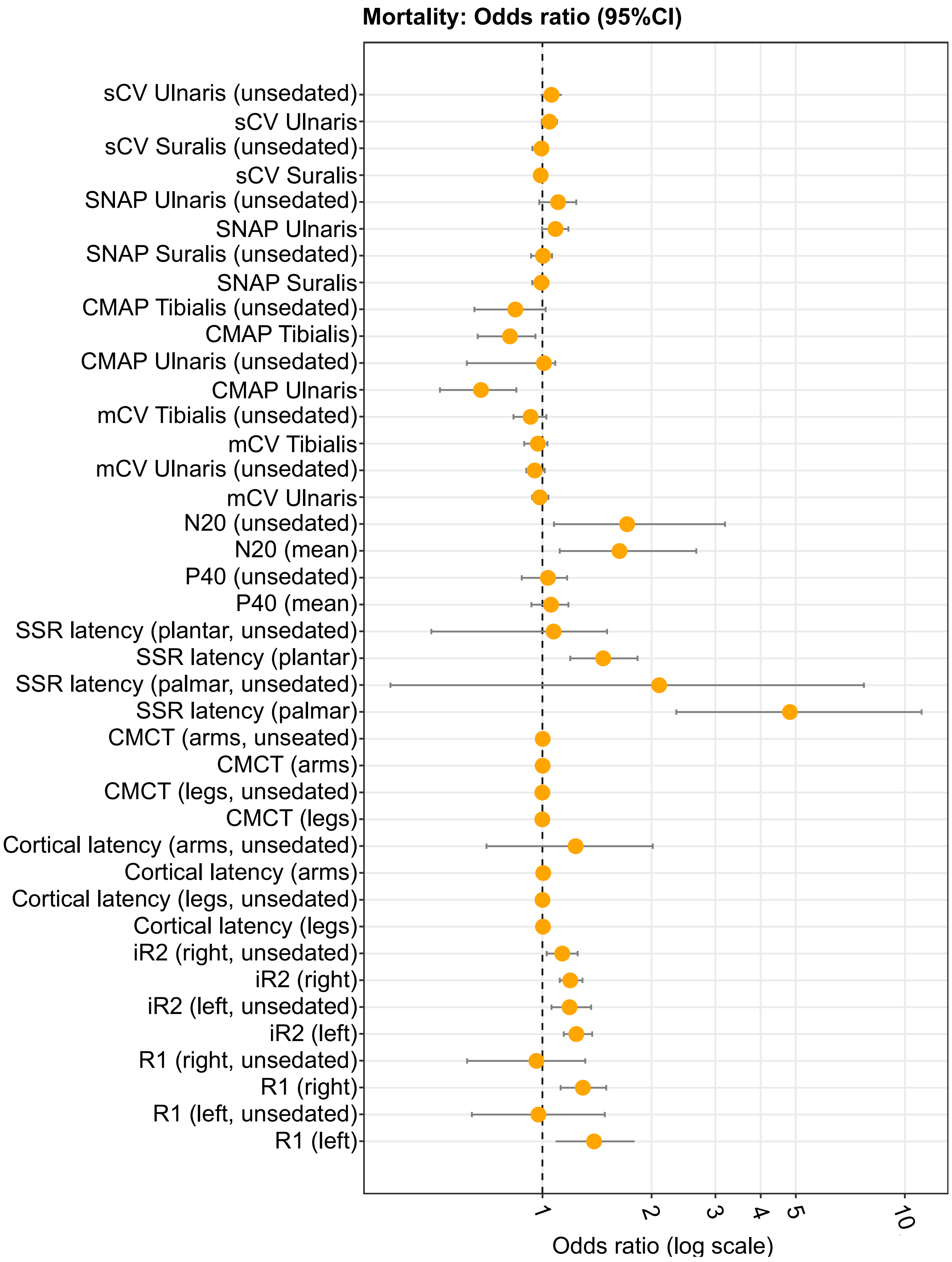
Mortality: Odds ratio (95%CI)
sCV Ulnaris (unsedated)
sCV Ulnaris
sCV Suralis (unsedated)
sCV Suralis
SNAP Ulnaris (unsedated)
SNAP Ulnaris
SNAP Suralis (unsedated)
SNAP Suralis
CMAP Tibialis (unsedated)
CMAP Tibialis)
CMAP Ulnaris (unsedated)
CMAP Ulnaris
mCV Tibialis (unsedated)
mCV Tibialis
mCV Ulnaris (unsedated)
mCV Ulnaris
N20 (unsedated)
N20 (mean)
P40 (unsedated)
P40 (mean)
SSR latency (plantar, unsedated)
SSR latency (plantar)
SSR latency (palmar, unsedated)
SSR latency (palmar)
CMCT (arms, unseated)
CMCT (arms)
CMCT (legs, unsedated)
CMCT (legs)
Cortical latency (arms, unsedated)
Cortical latency (arms)
Cortical latency (legs, unsedated)
Cortical latency (legs)
iR2 (right, unsedated)
iR2 (right)
iR2 (left, unsedated)
iR2 (left)
R1 (right, unsedated)
R1 (right)
R1 (left, unsedated)
R1 (left)
1
2
3
4
5
10
Odds ratio (log scale)

Supplementary Figure S4 Odds Ratios (95% CI) of mortality excluding patients with diabetes mellitus

This figure shows the odds ratios of mortality (with 95% confidence interval) for the subsample of patients without diabetes mellitus on the log scale. The number of comorbidities was defined according to the extended Charlson comorbidity index.[9] All scores refer to the time of the examination if not indicated otherwise. *p*-values < .05 are in boldface.

DM = diabetes mellitus, CI = confidence interval, MRS = Modified Rankin Scale, PNS = peripheral nervous system, NCS = nerve conduction studies, MEP = motor evoked potentials, SSEP = somatosensory evoked potentials, SSR = sympathetic skin response, BR = blink reflex.

MRS: 2.58 (1.23-5.71), p=**.012**; Number of comorbidities: 1.53 (0.74-2.81), p=.23; Male sex: 2.25 (0.45-22.04), p=.35; Age: 1.03 (0.98-1.07), p=.25; Lymphocytes: 1.09 (0.00-1.38), p=.64; C-reactive protein: 1.17 (1.06-1.33), p=**.00037**; Urea concentration: 1.02 (1.00-1.03), p=**.040**; Oxygen saturation: 0.81 (0.70-0.90), p**<.0001**; Respiratory rate: 1.04 (0.82-1.19), p=.71; Radiographic infiltrates: 3.17 (0.61-31.48), p=.18; Oxygen therapy: 5.78 (1.33-33.29), p=**.019**; Sedation: 121.80 (15.82- 1668.83), p**<.0001**; WHO score ≥ 5 (unsedated): 1.10 (0.09- 13.93), p=.93; WHO score ≥ 5 (all): 5.20 (1.00-51.66), p=.050; WHO score ≥ 6 (unsedated): 21.67 (1.54-313.38), p=**.026**; WHO score ≥ 6 (all): 74.74 (12.54-815.13), p**<.0001**; WHO score ≥ 7 (all): 165.00 (21.49-2257.18), p**<.0001**; PNS affection (unsedated): 3.05 (0.24-424.99), p=.43; PNS affection (all): 8.22 (0.93-1082.50), p=.061; CNS affection (unsedated): 13.00 (1.02-1814.60), p=**.049**; CNS affection (all): 4.64 (0.97-27.88), p=.054; NCS Pathology (unsedated): 2.97 (0.23-413.73), p=.44; NCS Pathology (all): 7.59 (0.86-1000.89), p=.074; MEP Pathology (unsedated): 2.64 (0.20-34.26), p=.42; MEP Pathology (all): 6.07 (0.92- 65.93), p=.061; SSEP Pathology (unsedated): 12.37 (0.90-1770.22), p=.061; SSEP Pathology (all): 6.02 (0.86-68.41), p=.071; SSR Pathology (unsedated): 4.03 (0.31-52.11), p=.26; SSR Pathology (all): 13.93 (2.55-142.51), p=**.0019**; BR Pathology (unsedated): 10.29 (0.77-138.07), p=.07; BR Pathology (all): 33.77 (5.91-356.40), p**<.0001**

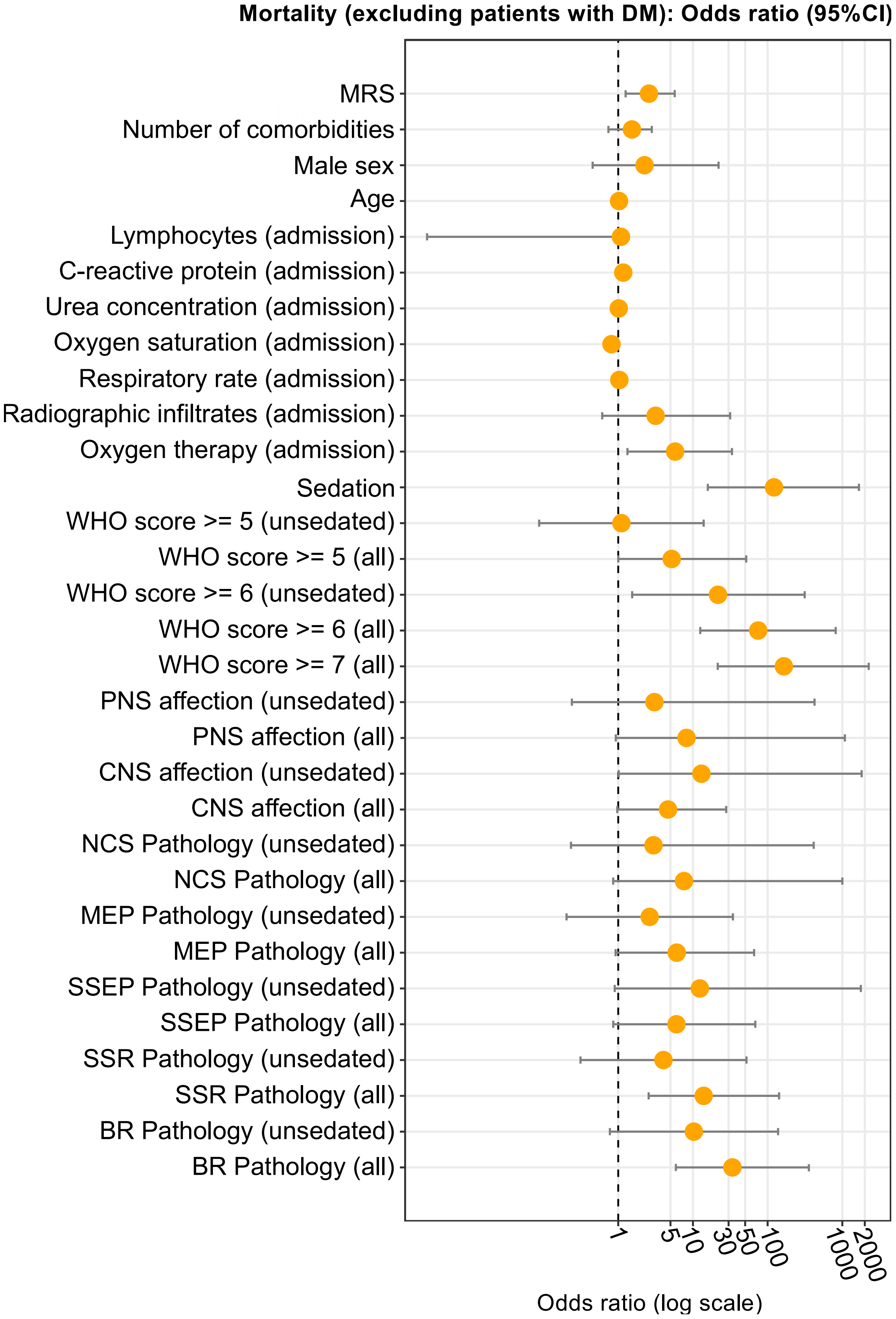
Mortality (excluding patients with DM): Odds ratio (95%CI)
MRS
Number of comorbidities
Male sex
Age
Lymphocytes (admission)
C-reactive protein (admission)
Urea concentration (admission)
Oxygen saturation (admission)
Respiratory rate (admission)
Radiographic infiltrates (admission)
Oxygen therapy (admission)
Sedation
WHO score >= 5 (unsedated)
WHO score >= 5 (all)
WHO score >= 6 (unsedated)
WHO score >= 6 (all)
WHO score >= 7 (all)
PNS affection (unsedated)
PNS affection (all)
CNS affection (unsedated)
CNS affection (all)
NCS Pathology (unsedated)
NCS Pathology (all)
MEP Pathology (unsedated)
MEP Pathology (all)
SSEP Pathology (unsedated)
SSEP Pathology (all)
SSR Pathology (unsedated)
SSR Pathology (all)
BR Pathology (unsedated)
BR Pathology (all)
1
5
10
30
50
100
1000
2000
Odds ratio (log scale)

Supplementary Figure S5 Odds Ratios (95% CI) of mortality based on the raw values in the electrophysiological assessment excluding patients with diabetes mellitus

This figure shows the odds ratios of mortality (with 95% confidence interval) on the log scale, based on the raw values in the electrophysiological assessment, for the subsample of patients without diabetes mellitus. The mean of the left and right sight of the body was used for the N20, P40, CMCT and cortical latency. *p*-values < .05 are in boldface.

DM = diabetes mellitus, CI = confidence interval, sCV = sensory nerve conduction velocity, SNAP = sensory nerve action potential, CMAP = compound muscle action potential, mCV = motor nerve conduction velocity, SSR = sympathetic skin response, CMCT = central motor conduction time.

sCV Ulnaris (unsedated): 0.89 (0.67-1.07), p=.36; sCV Ulnaris: 0.98 (0.88-1.06), p=.68; sCV Suralis (unsedated): 0.97 (0.73-1.06), p=.63; sCV Suralis: 0.99 (0.93-1.04), p=.71; SNAP Ulnaris (unsedated): 0.69 (0.32-1.12), p=.28; SNAP Ulnaris: 0.99 (0.80-1.11), p=.84; SNAP Suralis (unsedated):0.97 (0.00-1.09), p=.73; SNAP Suralis: 0.99 (0.90-1.05), p=.77; CMAP Tibialis (unsedated): 0.67 (0.32-1.03), p=.075; CMAP Tibialis: 0.71 (0.49-0.92), p=**.0055**; CMAP Ulnaris (unsedated): 1.00 (0.63-1.65), p>.99; CMAP Ulnaris: 0.68 (0.49-0.90), p=**.0065**; mCV Tibialis (unsedated): 0.90 (0.80-1.06), p=.25; mCV Tibialis: 0.98 (0.87-1.06), p=.75; mCV Ulnaris (unsedated): 0.89 (0.71-1.06), p=.27; mCV Ulnaris: 1.01 (0.91-1.07), p=.90; N20 (unsedated): 3.29 (1.09-2941.67), p=**.033**; N20 (mean): 2.75 (1.33-8.29), p=**.0043**; P40 (unsedated): 1.01 (0.69-1.18), p=.89; P40 (mean): 1.05 (0.90-1.18), p=.51; SSR latency (plantar, unsedated): 1.23 (0.59-1.82), p=.42; SSR latency (plantar): 1.61 (1.25-2.18), p=**.00029**; SSR latency (palmar, unsedated): 2.04 (0.09-10.23), p=.57; SSR latency (palmar): 5.87 (2.46-16.73), p**<.0001**; CMCT (arms, unsedated): 1.00 (1.00-1.01), p=.24; CMCT (arms): 1.00 (1.00-1.01), p=.32; CMCT (legs, unsedated): 1.00 (0.99-1.00), p=.96; CMCT (legs): 1.0 (0.99-1.00), p=.99; Cortical latency (arms, unsedated): 1.61 (0.78-3.26), p=.16; Cortical latency (arms): 1.01 (1.00-1.01), p=**.0012**; Cortical latency (legs, unsedated): 1.00 (1.00-1.01), p=.34; Cortical latency (legs): 1.00 (1.00-1.01), p=**.0063**; iR2 (right, unsedated): 1.22 (1.07-1.44), p=**.0044**; iR2 (right): 1.27 (1.15-1.45), p**<.0001**; iR2 (left, unsedated): 1.23 (1.07-1.46), p=**.0061**; iR2 (left): 1.28 (1.16-1.49), p**<.0001**; R1 (right, unsedated): 1.28 (0.86-1.72), p=.17; R1 (right): 1.37 (1.15- 1.68), p=**.00072**; R1 (left, unsedated): 1.33 (0.65-2.56), p=.44; R1 (left): 1.46 (1.04-2.07), p=.029

Mortality (excluding patients with DM) based on raw values: Odds ratio (95%CI)

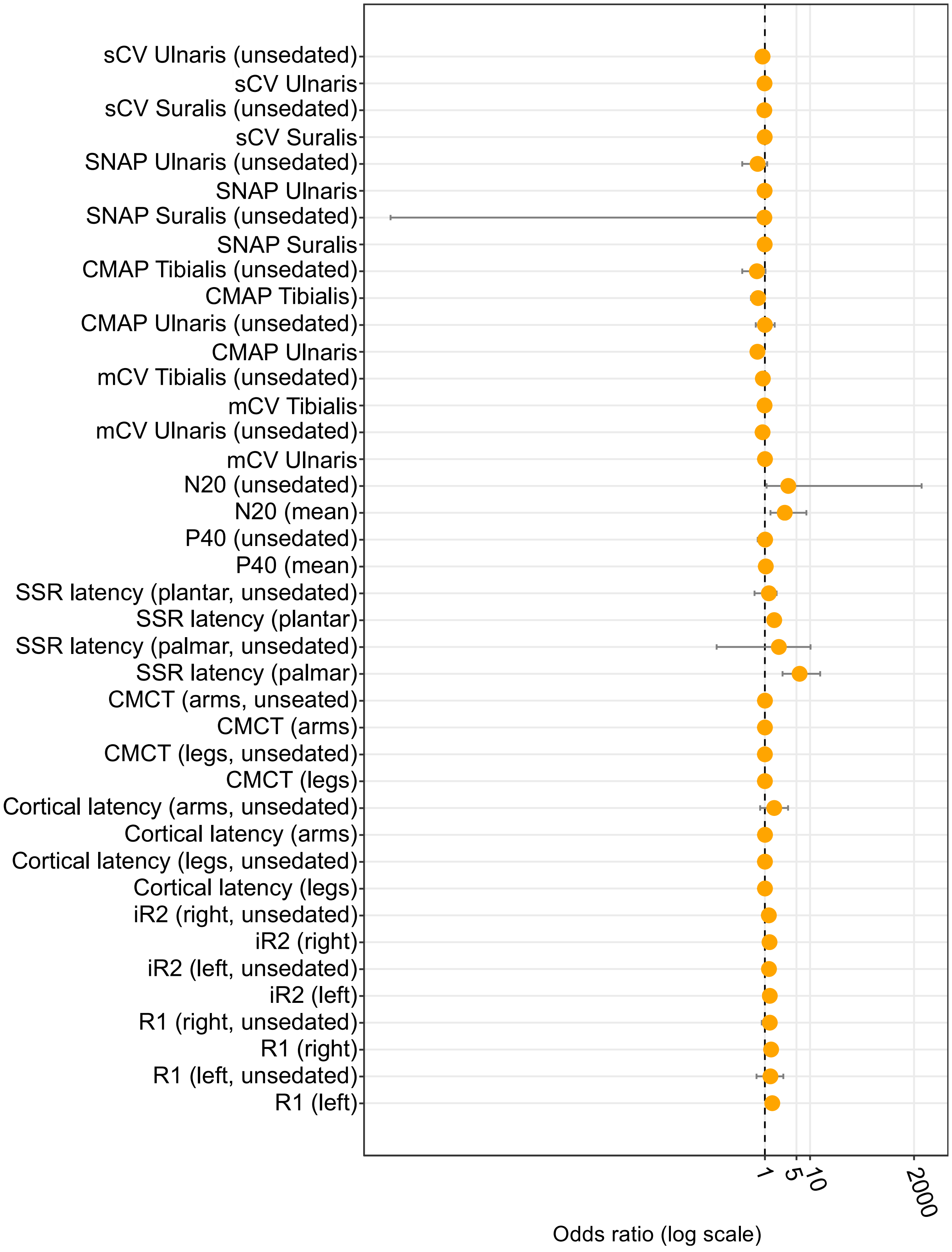

Supplementary Figure S6 Kaplan-Meier-Curves and Cox proportional hazard ratios for each electrophysiological assessment including and excluding sedated patients

This figure illustrates the Kaplan-Meier-Curves, Cox proportional hazard ratios and number of remaining patients under observation for all electrophysiological assessments from the time of assessment until discharge. Data are presented including and excluding sedated patients.

A) All patients that underwent SSEP assessment; B) All unsedated patients that underwent SSEP assessment; C) All patients that underwent MEP assessment; D) All unsedated patients that underwent MEP assessment; E) All patients that underwent BR assessment; F) All unsedated patients that underwent BR assessment; G) All patients that underwent NCS assessment; H) All unsedated patients that underwent NCS assessment; I) All patients that underwent SSR assessment; J) All unsedated patients that underwent SSR assessment; K) All patients that underwent any assessment of the PNS; L) All unsedated patients that underwent any assessment of the PNS; M) All patients that underwent any assessment of the CNS; N) All unsedated patients that underwent any assessment of the CNS; O) Percent of discharged patients in relation to the time of hospitalization.

SSEP = somatosensory evoked potentials, MEP = motor evoked potentials, BR = blink reflex, NCS = nerve conduction studies, SSR = sympathetic skin response, PNS = peripheral nervous system, CNS = central nervous system.

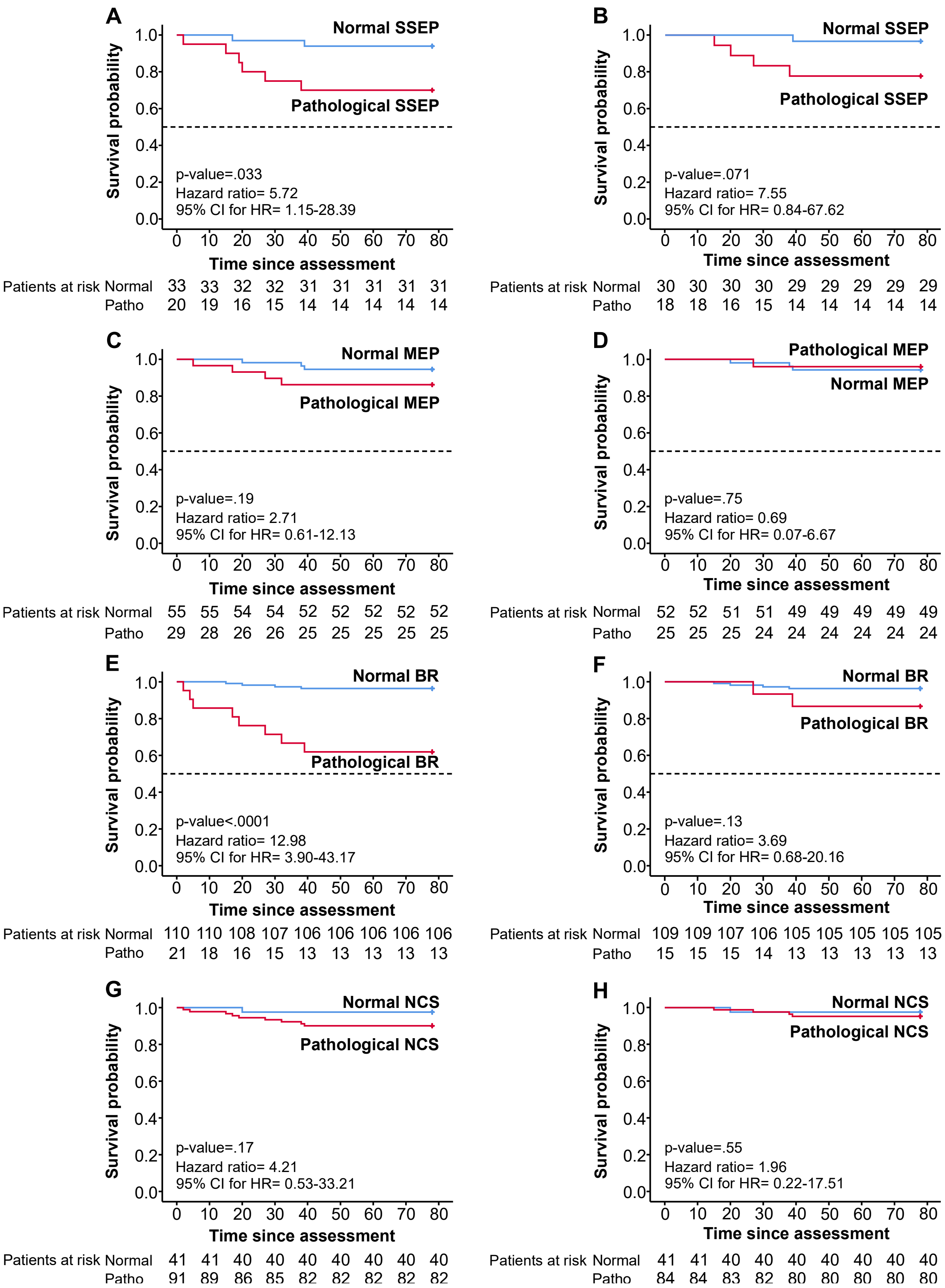
A
Normal SSEP
Pathological SSEP
p-value=.033
Hazard ratio= 5.72
95% CI for HR= 1.15-28.39
Survival probability
Time since assessment
Patients at risk Normal 33 33 32 32 31 31 31 31 31
Patho 20 19 16 15 14 14 14 14 14
B
Normal SSEP
Pathological SSEP
p-value=.071
Hazard ratio= 7.55
95% CI for HR= 0.84-67.62
Survival probability
Time since assessment
Patients at risk Normal 30 30 30 30 29 29 29 29 29
Patho 18 18 16 15 14 14 14 14 14
C
Normal MEP
Pathological MEP
p-value=.19
Hazard ratio= 2.71
95% CI for HR= 0.61-12.13
Survival probability
Time since assessment
Patients at risk Normal 55 55 54 54 52 52 52 52 52
Patho 29 28 26 26 25 25 25 25 25
D
Pathological MEP
Normal MEP
p-value=.75
Hazard ratio= 0.69
95% CI for HR= 0.07-6.67
Survival probability
Time since assessment
Patients at risk Normal 52 52 51 51 49 49 49 49 49
Patho 25 25 25 24 24 24 24 24 24
E
Normal BR
Pathological BR
p-value<.0001
Hazard ratio= 12.98
95% CI for HR= 3.90-43.17
Survival probability
Time since assessment
Patients at risk Normal 110 110 108 107 106 106 106 106 106
Patho 21 18 16 15 13 13 13 13 13
F
Normal BR
Pathological BR
p-value=.13
Hazard ratio= 3.69
95% CI for HR= 0.68-20.16
Survival probability
Time since assessment
Patients at risk Normal 109 109 107 106 105 105 105 105 105
Patho 15 15 15 14 13 13 13 13 13
G
Normal NCS
Pathological NCS
p-value=.17
Hazard ratio= 4.21
95% CI for HR= 0.53-33.21
Survival probability
Time since assessment
Patients at risk Normal 41 41 40 40 40 40 40 40 40
Patho 91 89 86 85 82 82 82 82 82
H
Normal NCS
Pathological NCS
p-value=.55
Hazard ratio= 1.96
95% CI for HR= 0.22-17.51
Survival probability
Time since assessment
Patients at risk Normal 41 41 40 40 40 40 40 40 40
Patho 84 84 83 82 80 80 80 80 80

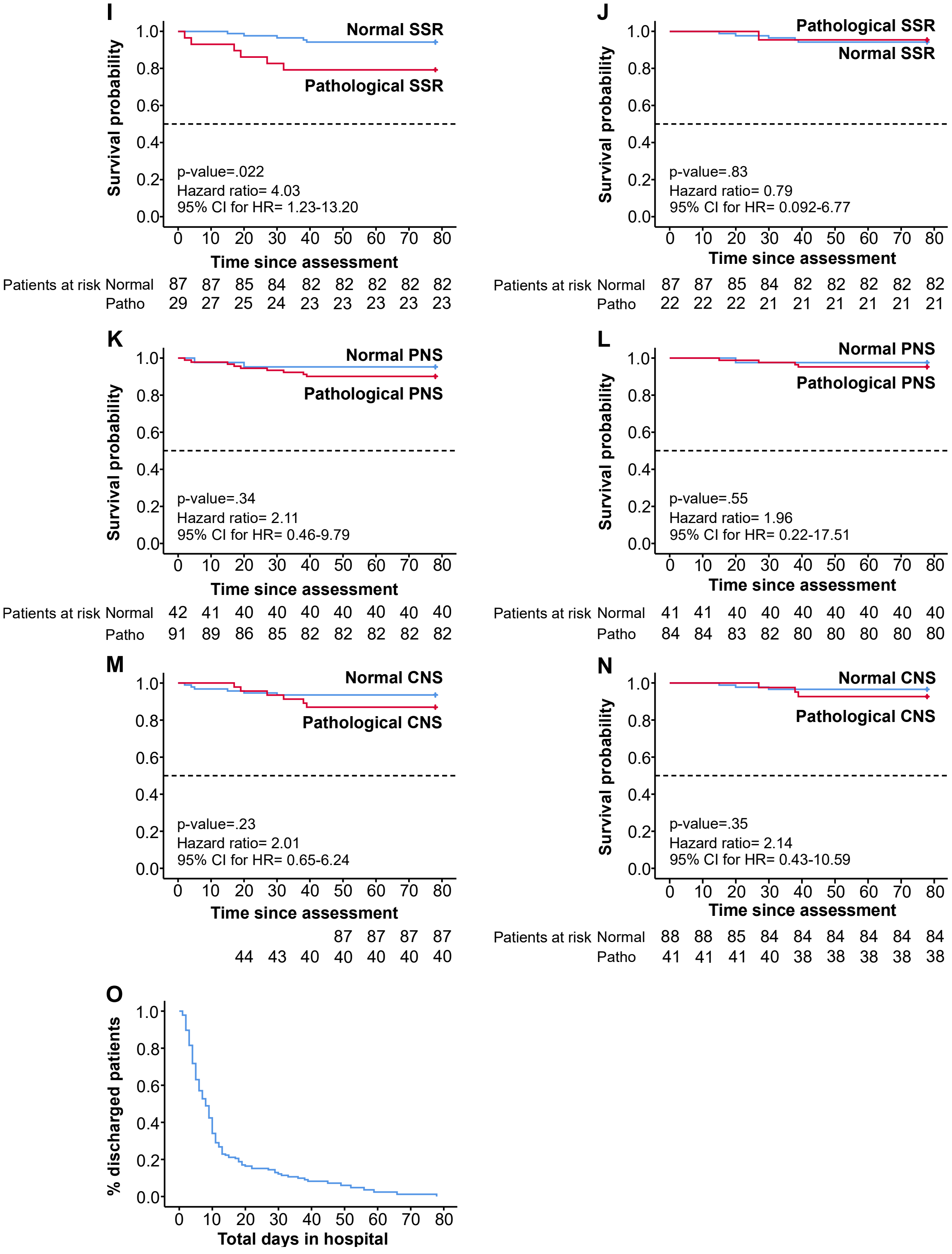
I
Normal SSR
Pathological SSR
p-value=.022
Hazard ratio= 4.03
95% CI for HR= 1.23-13.20
Survival probability
Time since assessment
Patients at risk Normal 87 87 85 84 82 82 82 82 82
Patho 29 27 25 24 23 23 23 23 23
J
Pathological SSR
Normal SSR
p-value=.83
Hazard ratio= 0.79
95% CI for HR= 0.092-6.77
Survival probability
Time since assessment
Patients at risk Normal 87 87 85 84 82 82 82 82 82
Patho 22 22 22 21 21 21 21 21 21
K
Normal PNS
Pathological PNS
p-value=.34
Hazard ratio= 2.11
95% CI for HR= 0.46-9.79
Survival probability
Time since assessment
Patients at risk Normal 42 41 40 40 40 40 40 40 40
Patho 91 89 86 85 82 82 82 82 82
L
Normal PNS
Pathological PNS
p-value=.55
Hazard ratio= 1.96
95% CI for HR= 0.22-17.51
Survival probability
Time since assessment
Patients at risk Normal 41 41 40 40 40 40 40 40 40
Patho 84 84 83 82 80 80 80 80 80
M
Normal CNS
Pathological CNS
p-value=.23
Hazard ratio= 2.01
95% CI for HR= 0.65-6.24
Time since assessment
87 87 87 87
44 43 40 40 40 40 40
N
Normal CNS
Pathological CNS
p-value=.35
Hazard ratio= 2.14
95% CI for HR= 0.43-10.59
Survival probability
Time since assessment
Patients at risk Normal 88 88 85 84 84 84 84 84 84
Patho 41 41 41 40 38 38 38 38 38
O
% discharged patients
Total days in hospital

References

1. Kurtzke JF. Rating neurologic impairment in multiple sclerosis: an expanded disability status scale (EDSS). *Neurology*. 1983;33(11):1444-1452. doi:10.1212/wnl.33.11.1444
2. van Swieten JC, Koudstaal PJ, Visser MC, Schouten HJ, van Gijn J. Interobserver agreement for the assessment of handicap in stroke patients. *Stroke*. 1988;19(5):604-607. doi:10.1161/01.STR.19.5.604
3. Hughes R, Bensa S, Willison H, et al. Randomized controlled trial of intravenous immunoglobulin versus oral prednisolone in chronic inflammatory demyelinating polyradiculoneuropathy. *Ann Neurol*. 2001;50(2):195-201. doi:10.1002/ana.1088
4. Mahoney FI, Barthel DW. Functional Evaluation: The Barthel Index: A simple index of independence useful in scoring improvement in the rehabilitation of the chronically ill. *Md State Med J*. 1965;14:61-65.
5. Scherer P, Baum K, Bauer H, Göhler H, Miltenburger C. Normierung der Brief Repeatable Battery of Neuropsychological Tests (BRB-N) für den deutschsprachigen Raum Anwendung bei schubförmig remittierenden und sekundär progredienten Multiple-Sklerose-Patienten. *Nervenarzt*. 2004;75(10):984-990. doi:10.1007/s00115-004-1729-0
6. Nasreddine ZS, Phillips NA, Bédirian V, et al. The Montreal Cognitive Assessment, MoCA: a brief screening tool for mild cognitive impairment. *J Am Geriatr Soc*. 2005;53(4):695-699. doi:10.1111/j.1532-5415.2005.53221.x
7. Thomann AE, Goettel N, Monsch RJ, et al. The Montreal Cognitive Assessment: Normative Data from a German-Speaking Cohort and Comparison with International Normative Samples. *J Alzheimers Dis*. 2018;64(2):643-655. doi:10.3233/JAD-180080
8. Fischer M, Kunkel A, Bublak P, et al. How reliable is the classification of cognitive impairment across different criteria in early and late stages of multiple sclerosis? *J Neurol Sci*. 2014;343(1-2):91-99. doi:10.1016/j.jns.2014.05.042
9. Gupta RK, Harrison EM, Ho A, et al. Development and validation of the ISARIC 4C Deterioration model for adults hospitalised with COVID-19: a prospective cohort study. *The Lancet Respiratory Medicine*. 2021;9(4):349-359. doi:10.1016/S2213-2600(20)30559-2
10. Teasdale G, Jennett B. ASSESSMENT OF COMA AND IMPAIRED CONSCIOUSNESS: A Practical Scale. *The Lancet*. 1974;304(7872):81-83. doi:10.1016/S0140-6736(74)91639-0
